# A natural biomimetic prosthetic hand with neuromorphic tactile sensing for precise and compliant grasping

**DOI:** 10.1126/sciadv.adr9300

**Published:** 2025-03-05

**Authors:** Sriramana Sankar, Wen-Yu Cheng, Jinghua Zhang, Ariel Slepyan, Mark M. Iskarous, Rebecca J. Greene, Rene DeBrabander, Junjun Chen, Arnav Gupta, Nitish V. Thakor

**Affiliations:** ^1^ Department of Biomedical Engineering, Johns Hopkins University, Baltimore, MD, USA.; ^2^ Department of Electrical Engineering and Computer Science, Florida Atlantic University, Boca Raton, FL, USA.; ^3^ Department of Electrical and Computer Engineering, Johns Hopkins University, Baltimore, MD, USA.; ^4^ Department of Biomedical Engineering, University of Illinois Chicago, Chicago, IL, USA.

## Abstract

The human hand’s hybrid structure combines soft and rigid anatomy to provide strength and compliance for versatile object grasping. Tactile sensing by skin mechanoreceptors enables precise and dynamic manipulation. Attempts to replicate the human hand have fallen short of a true biomimetic hybrid robotic hand with tactile sensing. We introduce a natural prosthetic hand composed of soft robotic joints and a rigid endoskeleton with three independent neuromorphic tactile sensing layers inspired by human physiology. Our innovative design capitalizes on the strengths of both soft and rigid robots, enabling the hybrid robotic hand to compliantly grasp numerous everyday objects of varying surface textures, weight, and compliance while differentiating them with 99.69% average classification accuracy. The hybrid robotic hand with multilayered tactile sensing achieved 98.38% average classification accuracy in a texture discrimination task, surpassing soft robotic and rigid prosthetic fingers. Controlled via electromyography, our transformative prosthetic hand allows individuals with upper-limb loss to grasp compliant objects with precise surface texture detection.

## Teaser

Biomimetic hybrid robotic prosthetic hand with tactile sensing precisely detects textures and compliantly grasps objects.

## INTRODUCTION

Soft robotics has grown over the past decade mainly due to its inherent compliance, increased safety in human-robot interaction, reduced cost, and increased dexterity (*1*, *2*). The compliant nature of soft robots makes them the better and safer choice for delicate object handling, directly interfacing with human bodies, and biomimicry of organisms and anatomical structures (*3*–*12*). Initially designed and developed as alternatives for traditional rigid robotics, soft robotic designs have recently expanded to unique applications by integrating sensors or multiple materials for robotic graspers and prostheses (*1*, *13*–*18*). However, soft robots composed of only compliant materials cannot consistently create the same grasping force to pick up heavy objects or palpate surface textures and objects as their rigid counterparts.

**Fig. 1.**
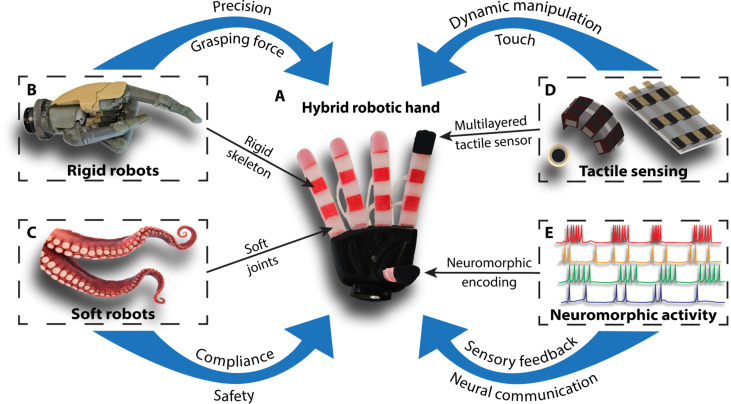
Hybrid robotic hand with neuromorphic tactile sensing inspired by the human hand. (**A**) Modeled on the human hand, the hybrid robotic hand combines the benefits of rigid and soft robots while adding tactile sensing and neuromorphic capabilities to manipulate objects safely and dexterously in unstructured environments. (**B**) Rigid robots, such as traditional prosthetic hands, provide precision and higher grasping forces, whereas (**C**) soft robots offer compliance and safety when interacting with objects. (**D**) Tactile sensing is necessary to manipulate and interact with objects dynamically. The hybrid biomimetic fingertip contains flexible multilayered tactile sensing inspired by the mechanoreceptors in human skin. (**E**) Neural communication efficiently sends large amounts of spatial and temporal sensory information to the brain. The hybrid robotic hand mimics the dynamic neural activity of nerves by neuromorphically encoding the tactile sensing information.

Human hands are hybrid structures that combine the precision and grasping force of rigid robots with the compliance and safety of soft robots (Fig. 1, B and C) (*19*). Many studies have tried to replicate the capabilities of the human hand with biomimetic rigid or soft robots (*7*, *8*, *20*–*25*). Of the few anthropomorphic hands with soft robotics, the maximum object weight they can lift is 1270 g (*26*, *27*). In addition, in the literature, none of the prosthetic hands with soft robotics or only partial hybrid robotic components have incorporated tactile sensing or neuromorphic encoding (*1*, *6*, *7*, *8*, *13*, *23*, *25*).

Tactile sensing is essential for interacting with objects in our environment and has been proven helpful for upper-limb prosthesis users to touch and dynamically manipulate objects during activities of daily living (Fig. 1D) (*28*). To understand and dynamically interact with our surroundings, humans receive static and dynamic cues from the mechanoreceptors in our skin (*29*). The four primary tactile mechanoreceptors are Merkel cells, Meissner corpuscles, Ruffini endings, and Pacinian corpuscles. In the outer epidermis layer of the skin, Merkel cells respond to light touch and Meissner corpuscles respond to low-frequency vibrations. In the deeper dermis layer of the skin, Ruffini endings detect deformation, and Pacinian corpuscles detect high-frequency vibrations and transient pressure. Using these mechanoreceptors, the human hand can sense diverse and complex surfaces. Simple static cues such as force and temperature can be sensed directly, but dynamic cues such as surface textures are feature rich and require more complicated processing using spatial and temporal tactile information. Texture discrimination is a benchmarking tool to assess a tactile sensing system’s ability to sense and differentiate various textured surfaces (*30*). Numerous studies have tested their tactile sensing on texture discrimination tasks, using 2 to 15 grated textures (*31*–*37*) or fewer natural textures (*38*–*43*). However, by not integrating these tactile sensors with soft or hybrid robots, they fail to harness the compliance advantage these robots provide.

Although there is an increasing trend toward incorporating sensing into soft robotics, most of these sensors focus on internal or simple tactile sensing of static cues (*1*, *6*, *13*, *44*–*49*). Integrating tactile sensors into soft robots is difficult because soft robotic actuation depends highly on their materials and construction. Adding rigid sensors to flexible and elastic soft robots would impair the soft robotic actuation or potentially cause material damage. Tactile sensing requires an increased and consistent flexion force while maintaining compliance. Therefore, integrating tactile sensors with soft or hybrid robots needs to prioritize flexibility and simple fabrication to maintain the normal function and compliance of the soft robotic components.

As tactile information is sensed, the spatial and temporal information from dynamic cues is encoded and sent to the brain through neural communication. Neural communication efficiently transmits large amounts of spatial and temporal information throughout our body using action potentials or spikes. Action potentials are event based, meaning they only signal when a change occurs. Thus, they reduce the load from multiple sensing inputs and subsequent processing of the combined spatial and temporal information. Humans can also perceive the same texture despite varying initial contact force at the receptor level. Therefore, for texture discrimination with neuromorphic encoding, the dynamic changes of the tactile sensor response across a textured surface are more important than the exact contact force measurement (*50*). The biomimetic approach of neuromorphic encoding increases the computational efficiency when processing the spatiotemporal information of textures and can provide naturalistic tactile sensory information to a prosthesis user through afferent nerve stimulation (*34*, *38*, *51*–*53*).

This work introduces a unique biomimetic hybrid robotic hand with embedded multilayered neuromorphic tactile sensing. First, we demonstrate an individual hybrid biomimetic finger of the hybrid robotic hand combining soft and rigid robotics with three independently actuated soft robotic joints and rigid endoskeleton. Second, the three layers of the multilayered biomimetic tactile sensor are embedded within the hybrid biomimetic finger’s fingertips with flexible sensing layers inspired by mechanoreceptors. Third, the neuromorphic encoding of the signals from each layer of the multilayered tactile sensor relative to the type of mechanoreceptors they represent to mimic dynamic neuronal activity. Fourth, we characterize the individual hybrid biomimetic finger from the hybrid robotic hand and multilayered tactile sensor to show the improved flexion force from the rigid endoskeleton while maintaining the benefits of compliance and safety features of soft robots. Fifth, we evaluate the multilayered tactile sensing in the individual hybrid biomimetic finger against a traditional rigid prosthetic finger and a pneumatically actuated soft robotic finger in a texture discrimination task involving palpating and discriminating 26 textured plates composed of soft and hard materials. Sixth, we validate the electromyography (EMG) controlled hybrid robotic hand’s grasping capability over various objects from the Southampton Hand Assessment Procedure (SHAP). Last, we demonstrate the hybrid robotic hand’s ability to sense and differentiate 15 everyday objects of varying surface textures and compliance during grasping. Our work pioneers biomimetic hybrid robotics with tactile sensing, resulting in an innovative biomimetic hybrid robotic prosthetic hand with embedded multilayered neuromorphic tactile sensing capable of object surface detection with compliant grasping.

## RESULTS

### Hybrid biomimetic finger

**Fig. 2.**
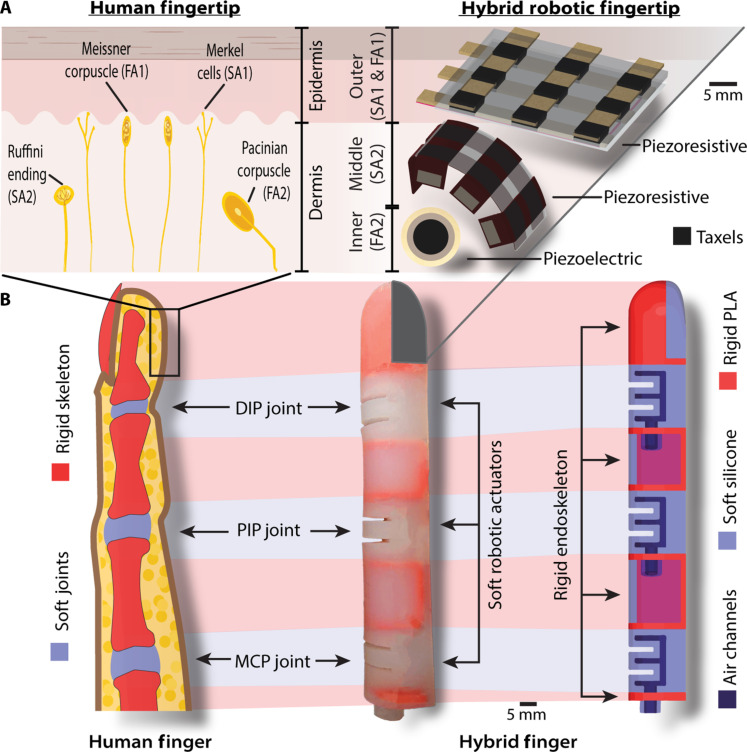
Hybrid finger with multilayered neuromorphic tactile sensing. (**A**) The mechanoreceptors in the epidermis and dermis of human skin detect and encode tactile information, inspiring the layers of tactile sensing in the hybrid fingertip. The layers of the tactile sensor are projected with an exploded view. The outer sensing layer on the surface of the hybrid fingertip represents the epidermis and contains a fabric 3 by 3 piezoresistive sensor with nine taxels (sensing elements). The middle and inner sensing layers represent the dermis. The middle layer is embedded in the silicone of the fingertip and contains a fabric 2 by 3 piezoresistive sensor with six taxels. The inner layer is adhered to the rigid PLA fingernail of the hybrid fingertip and contains a piezoelectric sensor. The multilayered tactile sensor mimics the neural encoding of the mechanoreceptors’ slow adapting (SA1&2) and fast adapting (FA1&2) receptors. (**B**) The hybrid finger components are inspired by the human finger, with the rigid PLA in red representing the endoskeleton and pneumatically actuated soft robotic actuators in blue representing the MCP, PIP, and DIP joints. The hybrid fingertip contains a rigid fingernail and a soft silicone fingertip to house the multilayered tactile sensor.

Inspired by the hybrid nature of the fingers in a human hand with soft and rigid anatomy, each hybrid biomimetic finger contains three independently actuated soft robotic joints made of Dragon Skin 10 silicone (Smooth-On, Easton, PA) placed between the rigid skeletal structures made of 3D printed polylactic acid (PLA) (Fig. 2). The hybrid robotic hand has 14 independently actuating joints, with three joints for each finger and two for the thumb. The soft robotic joints use pneumatic networks to actuate the hybrid finger by inflating when pressurized with air. The serial configuration of the soft actuators with the rigid endoskeleton provides a direct force transmission from the actuators to the manipulated object, thus increasing the flexion force of the hybrid finger compared to an entirely soft robotic finger (*24*).

The compliance of the hybrid biomimetic finger comes from its soft robotic joints and soft fingertip, allowing it to interact safely with objects. The soft actuator joints also allow for intrinsic joint actuation within the finger, eliminating the need for external tendons or motors. The fingertip of the hybrid biomimetic finger is constructed out of a rigid fingernail frame for structure and a soft silicone fingertip for compliance that contains the multilayered tactile sensor. The details of the hybrid finger fabrication process, pneumatic actuation system, and simplified kinematic model are provided in the Supplementary Materials (figs. S1 to S3).

### Multilayered biomimetic tactile sensor

Inspired by the layers of mechanoreceptors in human skin, the hybrid robotic hand’s fingertip contains three biomimetic layers of tactile sensors (Fig. 2). The tactile sensing layers were chosen based on their intrinsic characteristics to match the mechanoreceptors and their locations in the skin (*54*, *55*). Although some tactile sensors are constructed out of multiple layers, they do not have discrete sensors stacked on each other, especially with flexible sensing layers inspired by mechanoreceptors (*31*, *32*, *56*–*60*). The outer sensor layer represents the epidermis, with Merkel cells that respond to light touch and Meissner corpuscles that respond to low-frequency vibrations. Mimicking the dermis, the middle sensing layer represents the Ruffini endings that detect deformation and the inner sensing layer mimics the Pacinian corpuscles that detect high-frequency vibrations and transient pressure (*29*). The outer piezoresistive sensing layer on the surface of the hybrid fingertip has nine taxels (tactile sensing elements), each 4 mm^2^ and spaced 2.5 mm apart. The middle piezoresistive sensing layer embedded in the soft silicone of the hybrid fingertip has six taxels, each 6 mm^2^ and spaced 2.5 mm apart. Piezoresistive tactile sensors use materials that change electrical resistance when an external force is applied. The taxels on the middle sensing arrays have larger receptor fields and are offset from the outer layer to maximize sensing coverage across the hybrid fingertip.

The inner piezoelectric sensing layer is a 10-mm piezoelectric transducer (Uxcell, Hong Kong, China) adhered to the rigid fingernail between the soft and rigid components of the hybrid fingertip. Piezoelectric tactile sensors use materials that create a voltage when an external force is applied and only respond to the onset of forces. The unique multilayered tactile sensor was designed specifically for the hybrid finger to use the hybrid fingertip structure to its advantage. The outer and middle layers are flexible piezoresistive tactile sensors constructed out of fabric as part of the compliant silicone section of the fingertip, whereas the rigid piezoelectric inner sensing layer is on the fingertip’s rigid section to pick up vibrations (Fig. 2).

### Neuromorphic encoding

**Fig. 3.**
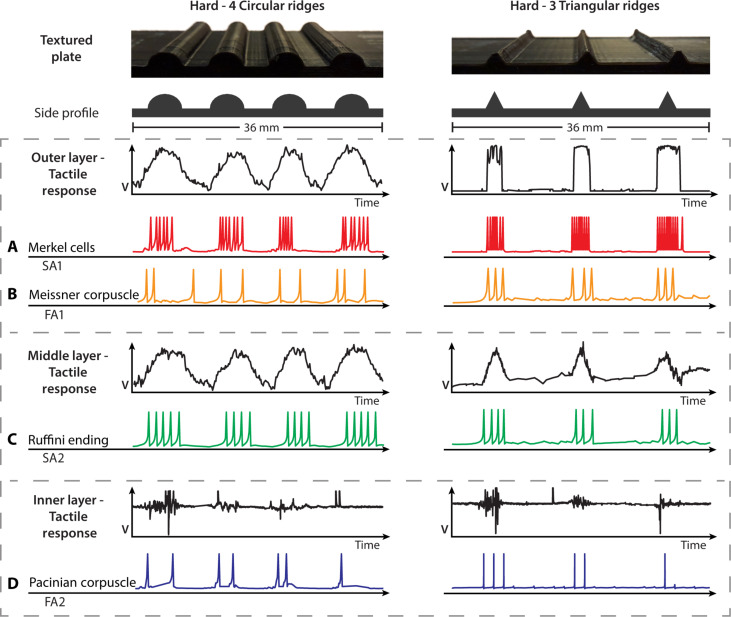
Neuromorphic responses of the sensing layers. The tactile responses from the “Hard - 4 circular ridges” and “Hard - 3 triangular ridges” textured plates for each sensing layer with their corresponding neuromorphically encoded responses. (**A**) Outer layer’s SA1 response for Merkel cells. (**B**) Outer layer’s FA1 response for Meissner corpuscles. (**C**) Middle layer’s SA2 response for Ruffini endings. (**D**) Inner layer’s FA2 response for Pacinian corpuscles.

The analog sensing response from each layer of the multilayer tactile sensor was neuromorphically encoded relative to the mechanoreceptors in human skin using the Izhikevich neuron model framework (*61*). Specific neuron models from this framework have independently worked well with texture discrimination, but we are using multiple neuron models to mimic the four primary mechanoreceptors, shown in further detail in Materials and Methods (*24*, *52*, *62*). Figure 3 shows the tactile and corresponding neuromorphic responses when the hybrid biomimetic finger with multilayered neuromorphic tactile sensor palpated two of the textured plates. Slowly adapting artificial Merkel cells and Ruffini endings continuously responding to a textured plate stimulus. The artificial Meissner corpuscle and Pacinian corpuscle have fast adapting (rapidly adapting) receptors and only respond at a stimulus’ onset or offset. Movie S1 shows the tactile response when palpating a textured plate with neuromorphic encoding in real time. The neuromorphic responses with machine learning are used to classify textures in this study but have been used to provide naturalistic tactile sensory information through nerve stimulation (*24*, *53*, *55*).

### Characterization

#### Finite element analysis

**Fig. 4.**
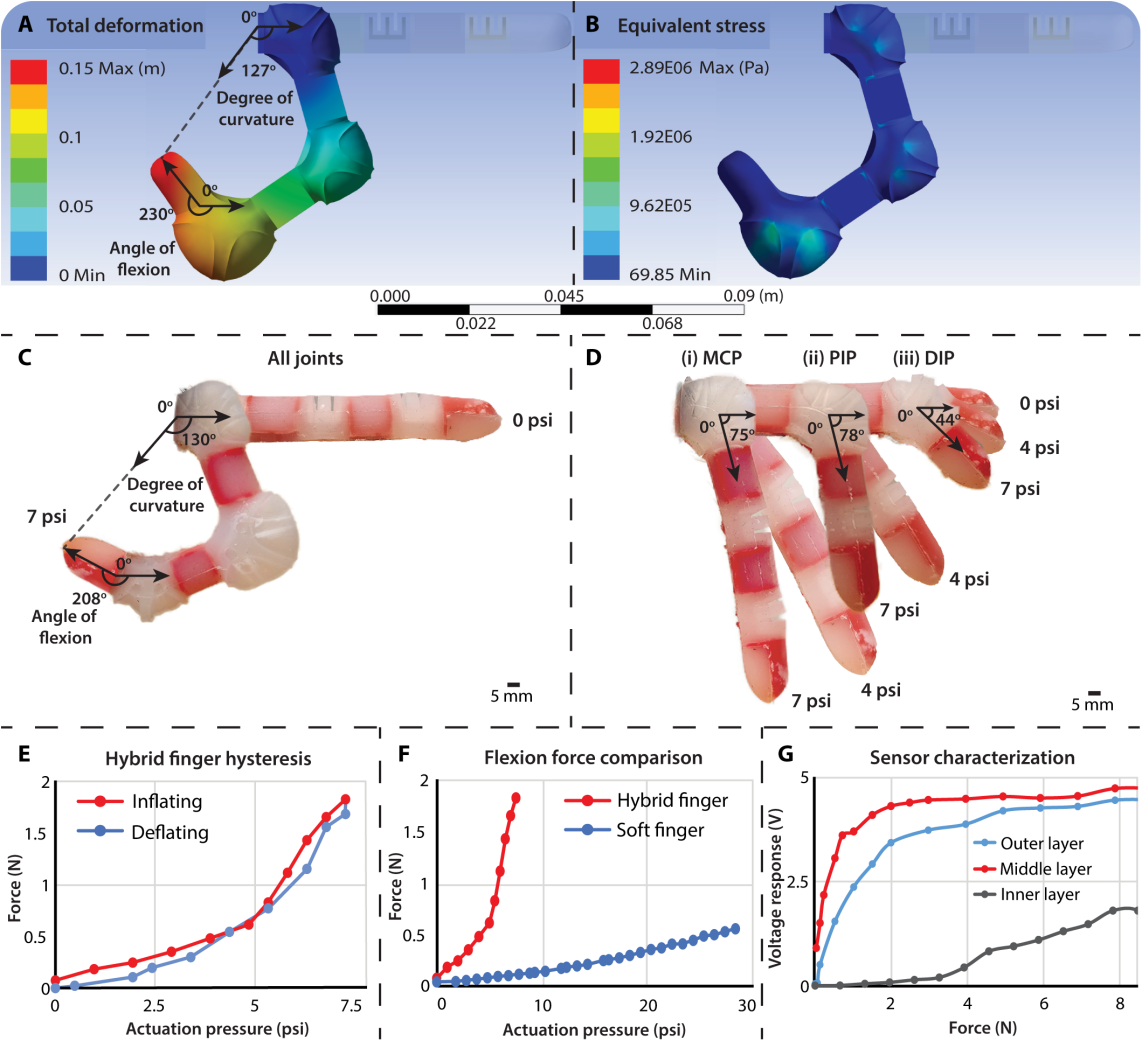
Hybrid finger and multilayered tactile sensor characterization. (**A**) The total deformation finite element analysis of the hybrid finger showed that the model achieved 127° of curvature and a 230° angle of flexion at 7 psi (48.26 kPa). (**B**) The equivalent stress finite element analysis of the hybrid finger during actuation showed no noticeable points of failure in the model. (**C**) The fabricated hybrid biomimetic finger achieved a degree of curvature of 130° and a 208° angle of flexion when all joints were actuated to 7 psi (48.26 kPa). (**D**) The actuation of the three independently actuated joints of the hybrid finger is shown at 0 psi (0 Pa), 4 psi (27.58 kPa), and 7 psi (48.26 kPa). The angle of flexion at 7 psi (48.26 kPa) at the (i) MCP joint was 75°, (ii) PIP joint was 78°, and (iii) DIP joint was 44°. (**E**) The hybrid finger could output a flexion force of 1.8 N when actuated to 7 psi (48.26 kPa) compared to the soft finger, which could only achieve 0.55 N at 28 psi (193.05 kPa). (**F**) The hybrid finger consistently actuates to 7 psi (48.26 kPa) with stable force application and minimal hysteresis even after 50 actuation cycles. (**G**) The characterization curve of all three layers of the tactile sensor showing sensor response based on the force applied to each sensor taxel. Each curve shows the average of all the sensing elements of that layer. The outer and middle layers are piezoresistive sensors, whereas the inner sensor layer is a commercial piezoelectric sensor that only responds to the onset of force.

To guide the hybrid robotic hand’s design process and evaluate the final design, finite element analysis was done on an individual hybrid biomimetic finger of the hybrid robotic hand using the ANSYS Discovery AIM Workbench (ANSYS, Canonsburg, PA). In the static structural analysis, standard PLA material data and a hyperelastic third-order Yeoh model of Dragon Skin were applied to the hybrid finger’s rigid and soft components. All three actuators in the hybrid finger were actuated at 7 psi (48.26 kPa) with the total deformation and equivalent von Mises stress shown in (Fig. 4, A and B). The hybrid biomimetic finger achieved 127° of curvature and a 230° angle of flexion with no points of failure. The degree of curvature measures the circular arc achieved by the tip of the finger, whereas the flexion angle is the change in angle between the planes of the fingertip and the base of the finger.

#### Individual hybrid biomimetic finger actuation

The angle of flexion and degree of curvature of an individual hybrid biomimetic finger from the hybrid robotic hand were measured at varying levels of actuating pressure (Fig. 4, C and D). The hybrid biomimetic finger achieved a degree of curvature of 130° and a 208° angle of flexion when actuated to 7 psi (48.26 kPa). Comparatively, the pneumatically actuated soft robotic finger only achieved 85° of curvature and a 130° angle of flexion at 27 psi (186.15 kPa) above atmospheric pressure (*24*). The actuation of the physical hybrid biomimetic finger achieved a comparable deformation to the finite element analysis. In human fingers, the metacarpophalangeal (MCP) joint has a median angle of flexion of between 79° and 97°, the proximal interphalangeal (PIP) joint’s median is between 87° and 90°, and the distal interphalangeal (DIP) joint’s median is between 52° and 68° (*63*). The hybrid finger achieved similar angles of flexion at 7 psi (48.26 kPa), with the MCP joint at 75°, the PIP joint at 78°, and the DIP joint at 44°. The hybrid finger consistently actuates to 7 psi (48.26 kPa) with stable force application and minimal hysteresis even after 50 actuation cycles (Fig. 4E). Actuation pressures above 8 psi (55.16 kPa) resulted in larger hysteresis with actuator bursting occurring at pressures higher than 10 psi (68.95 kPa) due to material failure. Figure S4 shows the actuation of the soft finger, and movie S2 shows the actuation of the hybrid robotic hand and each hybrid finger.

#### Hybrid and soft finger flexion force comparison

The hybrid finger’s flexion force was compared to the soft robotic finger to see how much the addition of the rigid skeletal structure would ultimately affect the grasping force of the hybrid robotic hand (Fig. 4F) (*24*). The fingers were mounted on a UR5 robotic arm above a weighing scale to measure flexion force and actuated to varying levels (setup in fig. S5). No external force was applied to the fingers. The hybrid finger outputs more than three times the force of the soft finger, 1.8 N at 7 psi (48.26 kPa) compared to 0.55 N at 28 psi (193.05 kPa), at a quarter of the actuation pressure. However, a human finger can produce 32 ± 14 N, and rigid prosthetic fingers can produce about 34 N (*64*, *65*). The 3x increase in flexion force brings the hybrid finger one step closer to an ideal hybrid human hand for grasping and palpating objects while maintaining soft robotic compliance.

Another comparison of the flexion force that shows the benefit of the rigid endoskeleton was conducted by fabricating a hybrid finger with no endoskeleton (fig. S6). The comparison showed that the maximum flexion force the hybrid finger with no endoskeleton could achieve was 0.61 N when actuated to 7 N, much lower than the hybrid finger with an endoskeleton (1.8 N). Overall, the hybrid finger with no rigid endoskeleton had around the same flexion force as the soft robotic finger (0.55 N), with a lower actuation pressure. Therefore, the rigid endoskeleton adds to the flexion force of the hybrid finger, which ultimately affects the grasping force of the hybrid robotic hand.

#### Multilayered tactile sensor characterization

The multilayered tactile sensor’s voltage response to varying forces is shown in Fig. 4G. The raw analog sensor responses from the outer and middle piezoresistive layers before neuromorphic encoding were characterized independently. The inner piezoelectric sensor only responds to the onset of forces with a resonant frequency of 3 to 5 ± 0.5 kHz and a max resonant impedance of 300 ohms. The detection of high-frequency vibrations and transient pressures of the inner sensing layer is characteristic of Pacinian corpuscles (Fig. 3D).

The maximum frequency response of each sensing layer of the multilayered tactile sensor, indicating the transition from discrete to continuous, is shown in fig. S7A. The outer piezoresistive sensing layer reached 46 Hz, the middle piezoresistive sensing layer reached 40 Hz, and the inner piezoelectric sensing layer stayed discrete at 52 Hz. The testing setup was only capable of reaching 52 Hz. However, a fast Fourier transform of the inner sensor layer showed a high magnitude up to 500 Hz, which is half our tactile sensing system’s sampling rate of 1000 Hz. The stability of the multilayered tactile responses was tested with 1000 repetitions (fig. S7, B and C). The multilayered tactile sensor was stable for 1000 repetitions, with minor fluctuations in the middle sensing layer at the beginning and end.

### Texture classification performance from surface texture palpation

**Fig. 5.**
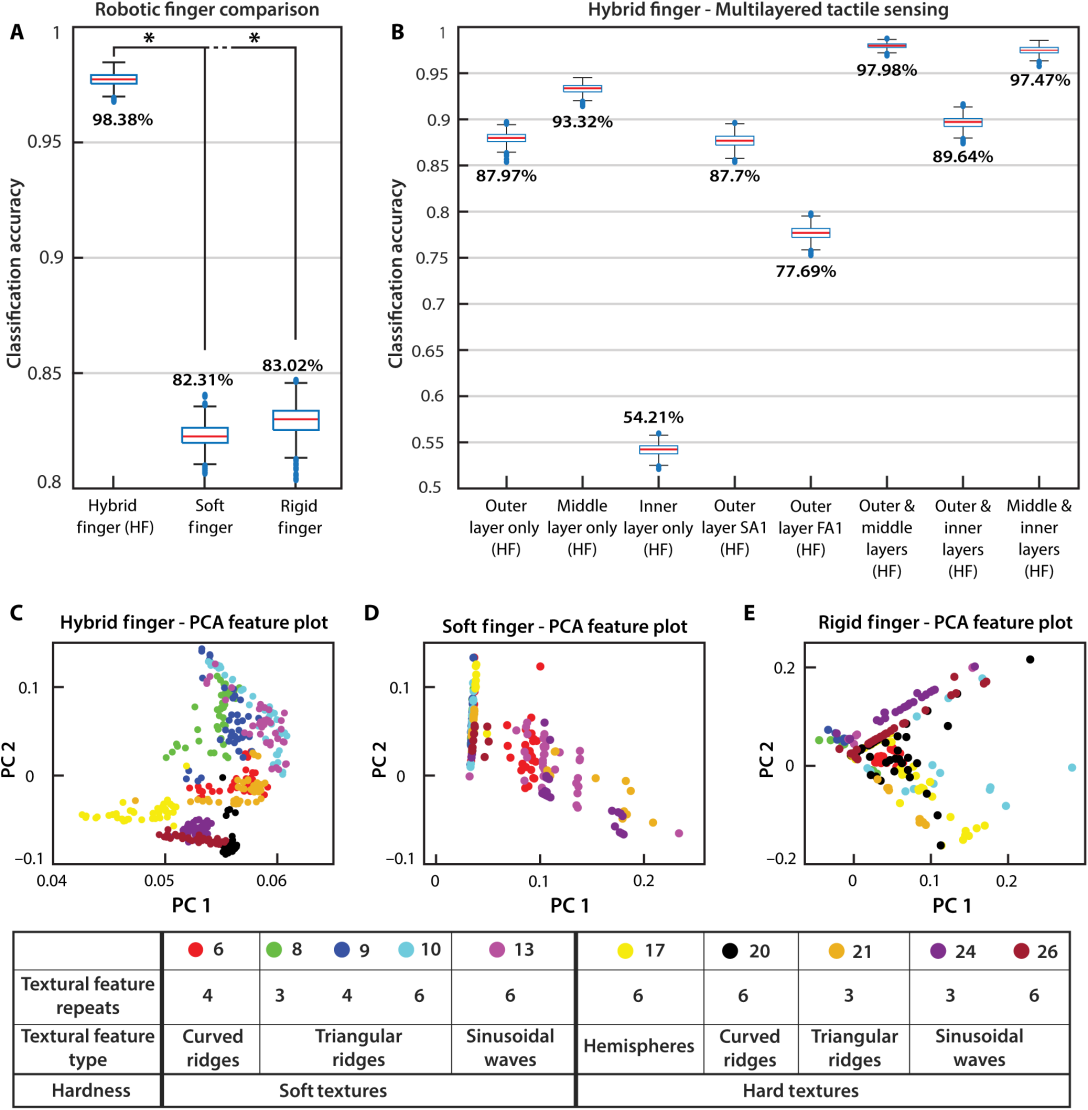
Texture classification accuracies. (**A**) The hybrid finger with multilayered sensing achieved an average classification accuracy of 98.38% when the classification was repeated a thousand times with *k*-fold cross-validation. The soft finger and rigid finger got 82.31 and 83.02%, respectively. One-way ANOVA revealed that there was a statistically significant difference (represented with *) in classification accuracy between the individual hybrid biomimetic finger and either soft finger (*P* value = 0) or rigid finger (*P* value = 0). Paired-samples *t* test showed a significant difference in texture classification accuracy between the individual hybrid biomimetic finger and either the soft finger (SD = 0.006, *P* value = 0) or rigid finger (SD = 0.0071, *P* value = 0) at a confidence level of 99%. (**B**) The individual sensing layers of the hybrid finger and their combinations were also assessed to show the benefits of multiple sensing layers. The hybrid finger with just the outer, middle, and inner sensing layers independently achieved average classification accuracies of 87.97, 93.32, and 54.21%. (**C** to **E**) Visualization of the feature space when plotting the first two principal components for the hybrid, soft, and rigid fingers. The classes with a classification accuracy lower than 75% were selected. The selected classes show the increased separation in texture classes of the hybrid finger compared to the other fingers, where the triangular ridges were stacked on top of each other. The quantitative separation between these clusters is represented by the overall texture classification accuracies of each finger. PCA, principal components analysis.

The texture discrimination capability of the hybrid robotic hand with multilayered neuromorphic tactile sensing was compared to the soft robotic finger and the rigid index finger of a Touch Bionics prosthetic hand (Össur, Orange County, CA). Only the outer tactile sensing layer was attached to the fingertips of the soft and rigid fingers because the middle and inner layers are intrinsic to the hybrid finger design. The additional sensing layers on the rigid prosthetic finger would require a complete redesign and cause interference with the soft finger’s actuation. The goal was to compare the hybrid finger to the soft and rigid fingers over the 26 textured plates to characterize and assess how accurately they could classify hard and soft textures. This experiment evaluates the finger’s ability to apply sufficient and consistent force on the textured surfaces while remaining compliant and the sensor’s ability to extract spatial and temporal information. The texture classification accuracies for the different fingers are provided in Fig. 5A, with corresponding confusion matrices provided in figs. S8 to S10.

The individual hybrid biomimetic finger with the multilayered tactile sensor successfully differentiated the 26 textured plates with 98.38% accuracy (fig. S8). The soft finger had an average classification accuracy of 82.31% but had trouble differentiating the soft triangular ridges and various hard textures with six textural repeats (fig. S9). The rigid finger had a similar average classification accuracy of 83.02% and had difficulty with soft textures, especially struggling with the soft and hard triangular ridges and soft sinusoidal waves (fig. S10). One-way analysis of variance (ANOVA) revealed that there was a statistically significant difference in classification accuracy between the individual hybrid biomimetic finger and either soft finger (*P* value = 0) or rigid finger (*P* value = 0). Paired-samples *t* tests showed a significant difference in texture classification accuracy between the individual hybrid biomimetic finger and either the soft finger (SD = 0.006, *P* value = 0) or rigid finger (SD = 0.0071, *P* value = 0) at a confidence level of 99%. Table S2 contains additional ANOVA metrics.

The texture discrimination task showed that the hybrid biomimetic finger could apply more consistent force over the textured plates than the soft finger while remaining more compliant than the rigid finger. Each layer of the tactile sensor and neuromorphically encoded models captured plenty of spatial and temporal information from the textures. The soft finger could not apply enough consistent force for the sensor to discern the smaller textural features, especially when there were multiple instances of the feature on the hard textured plates. The rigid finger is not compliant and applies too much force on the features of the textured plates, either crushing the soft textural features or saturating the tactile sensor response. Figure 5 (C to E) shows visualizations of the feature spaces when plotting the first two principal components for the hybrid, soft, and rigid fingers. The classes with a classification accuracy lower than 75% were selected. The selected classes show the increased separation in texture classes of the hybrid finger compared to the other fingers. The confusion matrices in figs. S8 to S10 show the types of textures the fingers had difficulty differentiating.

To justify the benefits of the multilayered sensor, the neuromorphic responses from each sensing layer were independently classified with multiple combinations. The classification accuracies for the layers of the multilayered sensor are provided in Fig. 5B, and the corresponding confusion matrices are provided in figs. S11 to S18. Compared to the classification accuracy of each layer independently, the multilayered biomimetic tactile sensor benefits from multiple sensing layers and neuromorphically encoded models. With just the outer layer sensor shared between fingers, the hybrid finger outperformed the soft and rigid finger at 87.97% average classification accuracy, compared to 82.31 and 83.02%, respectively. One-way ANOVA revealed that there was a statistically significant difference in classification accuracy between the individual hybrid biomimetic finger with only the outer sensing layer and either soft finger (*P* value = 0) or rigid finger (*P* value = 0). Paired-samples *t* tests showed a significant difference in texture classification accuracy between the individual hybrid biomimetic finger with only the outer sensing layer and either the soft finger (SD = 0.0084, *P* value = 0) or rigid finger (SD = 0.0092, *P* value = 0) at a confidence level of 99%. Table S2 contains additional ANOVA metrics.

The middle sensor layer performed the best and could pick up the soft triangular ridges that the other sensing layers had difficulty with. The outer sensing layer with only SA1 encoding performed well, with an 87.7% average classification accuracy, identical to the outer layer accuracy with both SA1 and FA1 encoding. The result suggests that RA1 encoding on the outer sensing layer does not bring in additional information from these textures. The sensing performance of the combined outer with middle layers and middle with inner layers was both nearly that of the full multilayered sensor, with a 97.98 and 97.47% average classification accuracy, respectively. The combined outer and inner layer’s sensing performance also increased to 89.64% average classification accuracy. The results indicate that each sensing layer brought varied textural information that increased the overall classification performance.

### Hybrid fingered hand

#### Grasping

**Fig. 6.**
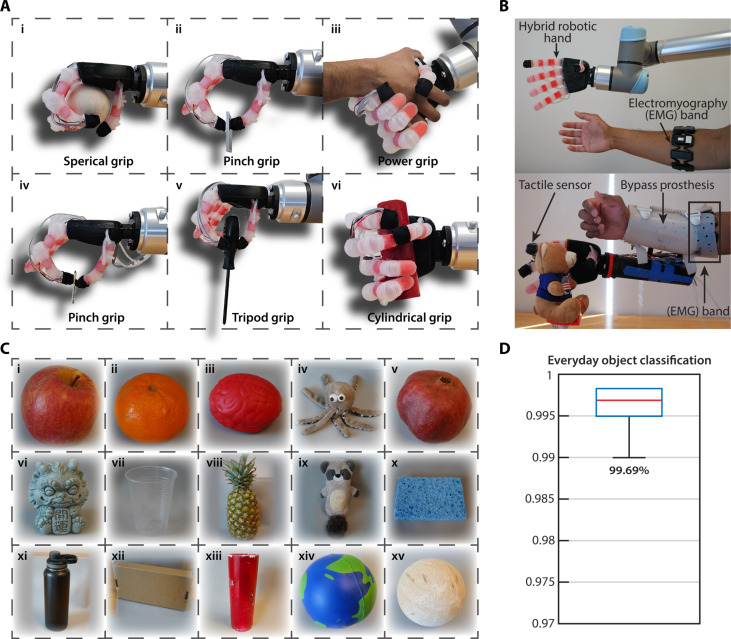
Hybrid robotic hand grasping and object identification capabilities. (**A**) Hybrid robotic hand grasping of everyday objects from the SHAP with different grip patterns: (i) spherical grip for a ball, (ii) pinch grip for a heavy 143-g metal plate, (iii) power grip for a handshake with a human hand, (iv) pinch grip for a delicate key, (v) tripod grip for a screwdriver, and (vi) cylindrical grip for a cylinder. (**B**) Hybrid robotic hand controlled by EMG on a bypass prosthesis. (**C**) Fifteen everyday objects that were differentiated using the multilayered tactile sensor of the hybrid robotic hand during grasping. (**D**) The hybrid robotic hand achieved an average classification of 99.69% when grasping and identifying the 15 everyday objects.

Once the performance and sensing capabilities of the individual hybrid biomimetic fingers were shown, the hybrid robotic hand was constructed from five fingers connected with a palm (Fig. 1). The index, middle, ring, and little fingers consist of four hybrid biomimetic fingers with three independently actuated joints each. The opposable thumb consists of a hybrid biomimetic finger with two independently actuated joints, following the anatomy of the human hand. An important goal for upper-limb prosthetic hands is to allow people with upper-limb loss to carry out activities of daily living involving a variety of grip patterns to grasp everyday objects. The hybrid robotic hand was attached to the UR5 robot arm and grasped objects from the SHAP (Fig. 6A). The SHAP is a hand function assessment for upper-limb prosthesis (*66*). Using the spherical grip, the hybrid hand can grasp a ball by conforming around the ball [Fig. 6A (i)], a grip pattern rigid prosthetic hands cannot properly achieve. The hybrid robotic hand also picked up a heavy metal plate weighing 143 g using the pinch grip with two fingers [Fig. 6A (ii)]. The increased flexion force of the hybrid fingers compared to a purely soft robotic finger leads to a higher grasping force. The hybrid hand is also capable of additional grip patterns, such as the power grip to perform a handshake with a human hand [Fig. 6A (iii)], pinch grip to grasp a key [Fig. 6A (iv)], tripod grip to grasp a screwdriver [Fig. 6A (v)], and cylindrical grip to grasp a cylinder [Fig. 6A (vi)]. The hybrid hand grasping and releasing objects are shown in movie S4.

The biomimetic hybrid robotic hand can also be controlled using EMG. People with upper-limb loss traditionally use EMG to control myoelectric prosthetic hands. The EMG signals are picked up and classified by the Myo Armband (Thalmic Labs, Ontario, CA). The hand positions are then sent to the Arduino microcontroller for the pneumatic actuation of the hybrid robotic hand (fig. S19). EMG signals from the Myo Armband are shown in figs. S20 and S21. In movie S4, we show the hybrid robotic hand controlled through EMG with a bypass prosthesis. The bypass prosthesis allows able-bodied subjects to use a prosthetic hand in experiments.

The hybrid hand can grasp delicate objects such as the plastic cup, stuffed toys, and fruit with compliance while also picking up heavy objects such as the filled metal water bottle (1600 g) (Fig. 6C). The best example of the hybrid robotic hand’s capability of grasping heavy objects with compliance is shown when it picks up a fragile plastic cup filled with water (280 g) using three fingers and without denting the cup (movie S4). Figure S22 shows a compilation of the heavy object grasping capability of the hybrid hand.

#### Everyday object differentiation during grasping

The multilayered tactile sensor in the fingertip of the hybrid robotic hand allows it to sense object surfaces and compliance while grasping. This was tested by the hand grasping 15 everyday objects of varying surface textures and compliance and differentiating them (Fig. 6C). The everyday objects included apple, clementine, foam brain, stuffed octopus toy, pomegranate, stone statue, plastic cup, pineapple, stuffed raccoon toy, dish sponge, metal water bottle, cardboard box, plastic cylinder, foam ball, and wood ball. The hand was mounted to the UR5 robotic arm, shown in Fig. 6A. The hybrid robotic hand achieved an average classification of 99.69% when grasping and identifying the 15 everyday objects (Fig. 6D) using the same texture discrimination method. On the basis of the confusion matrix, the only confusion came from the pineapple (fig. S23). The results indicate that each sensing layer brought varied textural information that increased the overall classification performance.

A visualization of the feature space when plotting the first two principal components based on the type of objects being grasped is shown in fig. S23. The objects were separated into fruits, compliant objects, and rigid objects. These feature plots show a good separation between objects of similar types and compliance. The classification accuracy of the individual sensing layers of the hybrid finger and their combinations was also assessed (fig. S24). Overall, these everyday objects were not complex for the multilayered tactile sensor to differentiate while grasping. The sensing layers that use the FA1 and FA2 encoding methods performed worse than the SA1 and SA2. This was especially noticeable in the outer layer SA1 at 99.31% compared to the FA1 at 91.57% using the same tactile sensor response. Therefore, these everyday objects require less vibration-based tactile sensing and more compliance-based sensing. Most everyday objects do not have minor surface variations that require a sensor with high frequency or sensitivity to differentiate. Combined with the compliance variation amongst objects, the ability to sense while grasping is essential for activities of daily living. However, tasks that require differentiation of minor surface variations would benefit from the inner piezoelectric sensor to measure the high-frequency vibrations during palpation. Therefore, the multilayered tactile sensor uses different sensing layers for various tasks, like the human fingertip. Thus, the multilayered tactile sensing of the hybrid robotic hand allows for compliant grasping and surface differentiation of everyday objects.

The biomimetic hybrid robotic hand presented here is capable of multiple grip patterns to grasp various objects of weight and shape using its increased flexion force and compliance. The embedded multilayered tactile sensor in the fingertips achieved exceptional tactile sensing capabilities over surface textures and everyday object differentiation during grasping. Overall, the biomimetic hybrid hand with embedded multilayered neuromorphic tactile sensing modeled on the human hand is a prosthetic hand for people with upper-limb loss to grant compliant object manipulation with elevated sensing and dexterity for activities of daily living.

## DISCUSSION

### Hybrid robotics

The field of soft robotics is growing due to the compliance benefits they have and their unique applications. However, catching up with established traditional rigid robotics requires considerable development to match their strength, precision, and resilience. As soft robots use active polymers to increase their strength and rigid robots use continuum mechanics to increase their degrees of freedom, they converge toward hybrid systems. This inevitable evolution into hybrid robotics has paralleled nature, with humans being an excellent hybrid structure consisting of a rigid skeletal frame controlled by soft muscle and tissue. Our study demonstrated the benefits of hybrid robotics by creating a biomimetic hybrid robotic hand. We mimicked the hybrid nature of a human finger by combining the compliance of a soft robot with the strength of a rigid endoskeleton to create biomimetic hybrid robotic fingers with three joints. Compared to the soft finger, the hybrid finger could output more than three times the flexion force of the soft finger at a quarter of the actuation pressure, 1.8 N at 7 psi (48.26 kPa) compared to 0.55 N at 28 psi (193.05 kPa). However, a human finger can produce 32 ± 14 N, and rigid prosthetic fingers can produce about 34 N. Although our approach to hybrid robotics builds on improving soft robotics, development is needed for the hybrid robotic hand to meet the force capabilities of the human hand or rigid prosthetics.

The individual hybrid biomimetic fingers achieved a degree of curvature of 130° and 230° flexion angle when actuated to 7 psi (48.26 kPa). Comparatively, the pneumatically actuated soft robotic finger only achieved an 85° of curvature and 130° flexion angle at 27 psi (186.15 kPa) above atmospheric pressure. The increased flexion force brings hybrid robotics one step closer to rigid robots for grasping and palpating objects while maintaining the essential compliance benefits of soft robots. The hybrid robotic hand can conform around and grasp objects using the spherical grip while having the strength to hold metal plates with the pinch grip, shown in the SHAP test. Using its increased flexion force and compliance, the biomimetic hybrid robotic hand is capable of multiple grip patterns to grasp objects of diverse weights and shapes.

### Sensing in soft robotics

The human hand is an excellent grasping tool because it incorporates a hybrid structure and includes somatosensation with proprioception to interact with our environment. To artificially replace the sense of touch, flexible and biomimetic sensing methods are a major endeavor because they can be adapted for various surfaces and applications. Tactile sensing is also important to protect the robot from potentially being damaged by the environment. However, even with the variety of flexible sensing, soft robots generally have unique architectures that do not allow them to work with traditional sensors because soft robotic actuation depends directly on its materials and construction. Therefore, tactile sensors need to be incorporated into soft robots without interfering with actuation. This requirement generally limits soft robots to internal strain sensing or simple extrinsic tactile sensing.

We designed a multilayered tactile sensor that uses the hybrid structure to its advantage without interfering with the actuation of the soft robotic actuators. The fingertip of the hybrid biomimetic finger is constructed out of a rigid fingernail frame for structure and a soft silicone fingertip for compliance. The flexible piezoresistive sensing arrays of the outer and middle layers provide tactile sensing as part of the compliant silicone section of the fingertip. The inner sensing layer is a rigid piezoelectric disc embedded on the fingertip’s rigid fingernail frame to pick up vibrations. The layered sensing approach mimics nature, including the deep vibration sensing of the Pacinian corpuscles. Now, the hybrid finger’s tactile sensing is focused on the fingertip to maximize surface contact during texture palpation, but additional sensors throughout the finger could be pursued.

### Neuromorphic encoding

The tactile sensor information is neuromorphically encoded to mimic the action potentials mechanoreceptors produce in the human hand. Neuromorphic encoding is advantageous for its biological relevance, computational efficiency, ability to encode various sensory stimuli, and potential for providing naturalistic sensory feedback to people with upper-limb loss through electrical nerve stimulation. The Izhikevich framework has limited similarity to receptor physiology compared to some neuromorphic frameworks, but the models we chose have been shown to work well with the complex task of texture discrimination. However, many models and parameters can be adjusted to create unique spiking patterns that follow the human physiology of mechanoreceptors and their encoding more closely to perform better. In this study, the neuromorphically encoded responses were used with machine learning to classify textures as a benchmark of the overall performance, but these neuromorphic signals have also been used to provide naturalistic tactile sensory information to prosthesis users through afferent nerve stimulation.

### Texture and object discrimination

For the texture discrimination task, the hybrid finger outperformed the soft and rigid fingers with a 98.38% average classification accuracy versus 82.31 and 83.02%, respectively. With just the sensory information from the outer layer of the multilayered sensor, the hybrid finger still achieved an average classification accuracy of 87.97%, signifying the benefits of the hybrid finger. With the same tactile sensor, the hybrid finger could apply more consistent force than the soft finger while maintaining compliance compared to the rigid finger. The soft finger had trouble differentiating textured surfaces with multiple instances of that textural feature, especially with hard textures due to a lack of enough consistent force applied across the entire textured plate. Adding more textured surfaces with multiple instances of that textural feature could better show the need for a higher consistent force for texture palpation. The rigid fingers had difficulty with many soft textures because the lack of compliance resulted in applying too much force on the textured plates, crushing the soft textural features, or saturating the tactile sensor. The piezoelectric sensor performed the worst at differentiating these textured surfaces but added to the sensing performance when combined with other layers. These textural elements do not properly test the vibration sensing of the piezoelectric sensor. Thus, adding more natural textured surfaces with varying hardness or natural surfaces could show the effects of a compliant and strong finger for texture palpation better. In addition, predicting the surface profiles of everyday objects. The large texture database from our data collection used a simple Support Vector Machine (SVM) classifier in this study as a benchmark. However, this database can test more intricate machine learning algorithms, such as convolutional and spiking neural networks.

The hybrid robotic hand achieved an average classification of 99.69% when grasping and differentiating the 15 everyday objects. Most rigid or soft robotic hands find these objects challenging to grasp, but the hybrid robotic hand’s flexion force and compliance allowed it to handle a variety of everyday objects without damaging them. The hybrid robotic hand’s multilayered tactile sensing additionally allows for surface differentiation of those everyday objects.

### Opportunities for future research

Providing sensory feedback to people with upper-limb loss requires sensors to pick up environmental information, neuromorphic encoding to translate that information into spike trains, and afferent nerve stimulation to deliver the sensation to the person. Although this work sets up the first two steps, transcutaneous electrical nerve stimulation has been used to provide textural information noninvasively. By bypassing the current texture classifier step, the spike trains produced by the neuromorphic encoding could be directly used to stimulate the user. Sensing during object manipulation of complex everyday objects shows the potential benefits of the hybrid robotic hand. Ultimately, a closed-loop prosthetic hand can be created by combining natural sensory feedback with the current hybrid robotic hand to actively sense and manipulate objects.

We use the human hand as a hybrid robotics model because of the versatile, general-purpose, and adaptable capabilities of the human hand. Although our biomimetic hybrid robotic hand had improved performance compared to soft and rigid fingers, it represents an early-stage prototype and does not leverage the full potential of hybrid robotics yet. The design of the hybrid biomimetic finger can be improved to have rigid linkages in the joints through the endoskeleton to increase structural integrity and flexion force compared to the current serial configuration. The additional flexion force will be beneficial in improving the capabilities of the hybrid robotic hand to achieve stronger grasping forces. The difficulty lies in adding a continuous rigid endoskeleton that does not interfere with the soft robotic actuation and compliance. The hybrid robotic hand with tactile sensing is a step toward an ideal replacement for a human hand to grasp objects with compliance and safely interact with the environment.

Combining two distinct ideas or technologies can result in a weaker option. However, we showed that incorporating a multilayered tactile sensor into a hybrid robot is better than the sum of its parts by achieving the highest classification accuracy. Each of the sensing layers brought in varied textural information that increased the overall performance of tactile sensing. Thus, this study demonstrates an innovative biomimetic hybrid robotic hand with multilayered tactile sensing and neuromorphic encoding modeled on the human hand capable of compliant grasping and manipulation with texture and object differentiation. Our work has the potential to create more natural prosthetic hands to allow people with upper-limb loss to sense and interact with their environment.

## MATERIALS AND METHODS

### Hybrid biomimetic finger

The hybrid biomimetic finger contains three independently actuated soft robotic joints made of Dragon Skin 10 silicone (Smooth-On, Easton, PA) between the rigid skeletal structures of 3D printed PLA. The length and overall volume of the hybrid finger were specified based on the average dimensions of an adult male human finger (82.68 mm) (*67*). Excluding the MCP joint, which connects the palm and fingers, the hybrid finger has a length of 85 mm (fig. S1). The length of the individual soft robotic joints was determined using the total deformation finite element analysis in ANSYS to achieve the total angle of flexion comparable to a human finger without any noticeable points of failure using the equivalent stress finite element analysis. In human fingers, the MCP joint has a median angle of flexion between 79° and 97°, the PIP joint’s median is between 87° and 90°, and the DIP joint’s median is between 52° and 68° (*63*). The length of each rigid component was determined by aligning the joints against the human finger and adding rigid components to connect the joints (Fig. 2). The actuation of the physical hybrid biomimetic finger achieved a comparable deformation to the finite element analysis. The hybrid finger achieved similar angles of flexion at 7 psi (48.26 kPa), with the MCP joint at 75°, the PIP joint at 78°, and the DIP joint at 44°.

The fingertip of the hybrid biomimetic finger is constructed out of a rigid fingernail frame for structure and a soft silicone fingertip for compliance that contains the multilayered tactile sensor. The fabrication process of the hybrid biomimetic fingers is shown in fig. S1. Silicone sealant is necessary to adhere the silicone actuating joints to the PLA endoskeleton. Because silicone is an elastomer and expands during actuation, it does not properly adhere to PLA unless the silicone sealant adhesive is used.

The pneumatic system to actuate a finger of the hybrid robotic hand is shown in fig. S2. It has a feedback loop with pressure sensors to ensure the pressure within each joint stays consistently at the targeted pressure. The pneumatic system used an air compressor, six two-way bidirectional miniature solenoid valves (Parker X-7-12-L-F), and pressure sensors (Honeywell ASDXACX100PAAA5) to regulate the airflow into each joint. However, this system can be simplified to use only three three-way direct-acting solenoid valves to regulate the air entering the joints from the compressor and releasing the air from the finger into the ambient atmosphere. An Arduino microcontroller independently controls the pneumatic circuit. Relays are used to control the 12V valves using the microcontroller. Each hybrid finger has three independently actuated joints, needing a scalable pneumatic system. However, the joints within each finger and other fingers on the hybrid robotic hand can be combined for simpler control.

### Multilayered tactile sensor

The outer and middle sensing layers were constructed by sandwiching conductive fabric traces (LessEMF, Latham, NY) in a grid pattern around piezoresistive fabric (Eonyx, Pinole, CA), with a fabric interface to adhere the layers together. A protective elastic fabric encases the outer sensing layer on the hybrid fingertip. The taxel sizes were based on the size of the conductive fabric traces, and the resolution was based on the limited space on the hybrid finger’s fingertip. Figure S25 shows an image of the three tactile sensing layers with exploded views of each layer. Figure S26 shows the data collection setup for the hybrid finger. The sensor responses were collected at a sampling rate of 1000 samples/s using an Arduino microcontroller. Voltage divider circuits with 1000-ohm resistors are connected in series with each common line of the piezoresistive sensors to measure the sensor responses. The piezoelectric sensor is connected to the Arduino through an operational amplifier (op-amp) with a gain of 1.51. Because the piezoelectric sensor has a relatively low voltage output with high impedance, the amplifier is necessary to work effectively.

### Neuromorphic encoding

The sensor response from each layer of the tactile sensor was neuromorphically encoded using the Izhikevich neuron model framework (*61*). The outer sensing layer mimicked the encoding of Merkel cells (SA1) and Meissner corpuscles (FA1) with the tonic spiking and phasic bursting models, respectively. The middle sensing layer mimicked the Ruffini endings (SA2) with the fast-spiking model, and the inner sensing layer mimicked the Pacinian corpuscle (FA2) with the phasic spiking model. These models were chosen because they follow a similar spiking pattern to the mechanoreceptors. The neuromorphic model generates a spike train with the normalized sensor output serving as the injected current *I*, recovery variable *u*, membrane voltage *v*, and gain *k* using Eqs. 1, 2, and 3. The parameters *a*, *b*, *c*, and *d* regulate the behavior of each spiking model and are shown in Table 1

$$\frac{\mathrm{dv}}{\mathrm{dt}}=0.04v^{2}+5v+140-u+\mathrm{kI}$$ (1)

$$\frac{\mathrm{du}}{\mathrm{dt}}=a\left(\mathrm{bv}-u\right)$$ (2)

$$\text{if}\;v\geq 30\;\mathrm{mv},\text{then}\;\left\{\begin{array}{c} v\leftarrow c\\ u\leftarrow u+d \end{array}\right.$$ (3)

**Table 1.** Neuronal model parameters.

	*a*	*b*	*c*	*d*	*k*
SA1	0.02	0.2	−65	8	75
FA1	0.02	0.25	−55	0.05	7
SA2	0.1	0.2	−65	2	75
FA2	0.02	0.25	−65	6	0.005

The continuous spike trains from the neuromorphic models were then compressed using two spike-based metrics, average interspike interval (Avg ISI) and average mean spike rate (Avg MSR). These spike-based metrics have been shown to pick up the special and temporal information from spike trains in human mechanoreceptors and artificial neuromorphic encoding strategies (*68*). The Avg ISI was calculated by measuring the time elapsed between each spike and averaging the values over the trial window. The AvgMSR was calculated by windowing the data into 100-ms bins, counting the spikes in each window, dividing that number by the 100-ms bin length, and averaging each MSR over the entire trial window. The Avg ISI and Avg MSR were calculated for the spike trains corresponding to each sensing element for every trial window.

### Experimental procedure for texture discrimination

**Fig. 7.**
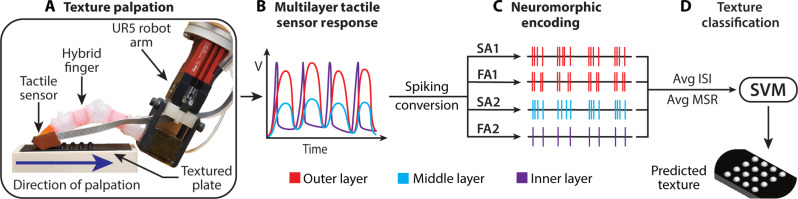
Experimental setup and procedure of texture discrimination using the hybrid finger. (**A**) Experimental setup showing the multilayer tactile sensor on the hybrid finger mounted on a UR5 robotic arm. The hybrid finger passively palpated the textured plates in the indicated direction. (**B**) Representation showing each sensor layer responds differently to the textured plates, with the outer and middle piezoresistive sensor layers responding to the continuous changes in textural elements and the inner piezoelectric sensor only responding to the initial occurrence of each textural element. (**C**) The sensor responses are then neuromorphically encoded relative to the skin’s mechanoreceptors, shown in Fig. 2, using the Izhikevich neuron model framework. The representation of the four resulting spike trains is shown. (**D**) Spike-based features, average interspike interval (Avg ISI) and average mean spike rate (Avg MSR), are used as inputs for the SVM classifier to differentiate between the textures and predict the palpated texture.

In this study, the hybrid, soft, and rigid fingers passively palpated the 26 textured plates in a texture discrimination task (fig. S27). This method evaluates the finger’s ability to apply consistent force and maintain compliance when identifying surface features without damaging those features or saturating the tactile sensor. The fingers were mounted on a UR5e Robot arm (Universal Robots, Novi, MI) to achieve consistent and repeatable texture data collection. The fingers were brought down on one side of the textured plate at 15° compared to the textured plate to allow the entire sensor surface to contact the textural features during palpation and applied a normal force of 1 N. Then, the fingers passively palpated the textured plate in the direction shown in Fig. 7. The hybrid finger was actuated to 7 psi (48.26 kPa) and the soft finger to 15 psi (103.42 kPa). Each of the 26 textured plates was passively palpated 40 times at ~64 mm/s. An overview of texture palpation is shown in movie S1.

### Textured plates

Texture discrimination is a benchmarking tool to access a tactile sensor’s ability to sense different surfaces. A total of 26 textured plates were made to assess and compare the hybrid, soft, and rigid fingers’ texture discrimination capabilities (fig. S27). Of the 26 textured plates, a set of 13 was 3D printed out of PLA and an identical set of 13 was made from Dragon Skin 10 silicone. The standardized 108 mm–by–36 mm textured plates had varying textural elements to assess the sensor’s ability to acquire spatial and temporal information (*24*). The varying hardness of the textured plate material assessed the finger’s ability to apply enough consistent force to identify surface features while maintaining compliance to not damage those features or saturate the tactile sensor. The centered 36 mm–by–36 mm textural features were raised to 2.5 mm above the textured plate.

### Texture classification

The spike-based metrics, Avg ISI and Avg MSR, from the neuromorphic encoding process were used as the input features for the machine learning classifier. Linear SVM was used because it has been shown to classify textures well, and the identities of the textures are known (*24*, *62*). *k*-fold cross-validation with a *k* = 4 was repeated a thousand times to reduce the bias. With a *k* = 4, the 40 trials are split randomly into four groups with 75% training and 25% testing, where the classifier is trained on three groups and then tested on the remaining group. The cross-validation process is completed four times, with each group used as the testing set. The final classification accuracy for each of the fingers and individual sensing layers is the average accuracy from all the runs of the *k*-fold cross-validation repeated a thousand times.
