# Supplementary material for: A natural biomimetic prosthetic hand with neuromorphic tactile sensing for precise and compliant grasping
Source: Sci Adv. 2025 Mar 5;11(10):eadr9300. doi: 10.1126/sciadv.adr9300 (PMC11881920; doi:10.1126/sciadv.adr9300)
Supplement: Supplementary file 1 — Figs. S1 to S27 Tables S1 and S2 Legends for movies S1 to S4 [file sciadv.adr9300_sm.pdf]

Science Advances
AAAS

Supplementary Materials for

A natural biomimetic prosthetic hand with neuromorphic tactile sensing for precise and compliant grasping

Sriramana Sankar *et al.*

Corresponding author: Sriramana Sankar, ssankar6@jhu.edu

Sci. Adv. **11**, eadr9300 (2025)
DOI: 10.1126/sciadv.adr9300

The PDF file includes:

Figs. S1 to S27
Tables S1 and S2
Legends for movies S1 to S4

Other Supplementary Material for this manuscript includes the following:

Movies S1 to S4

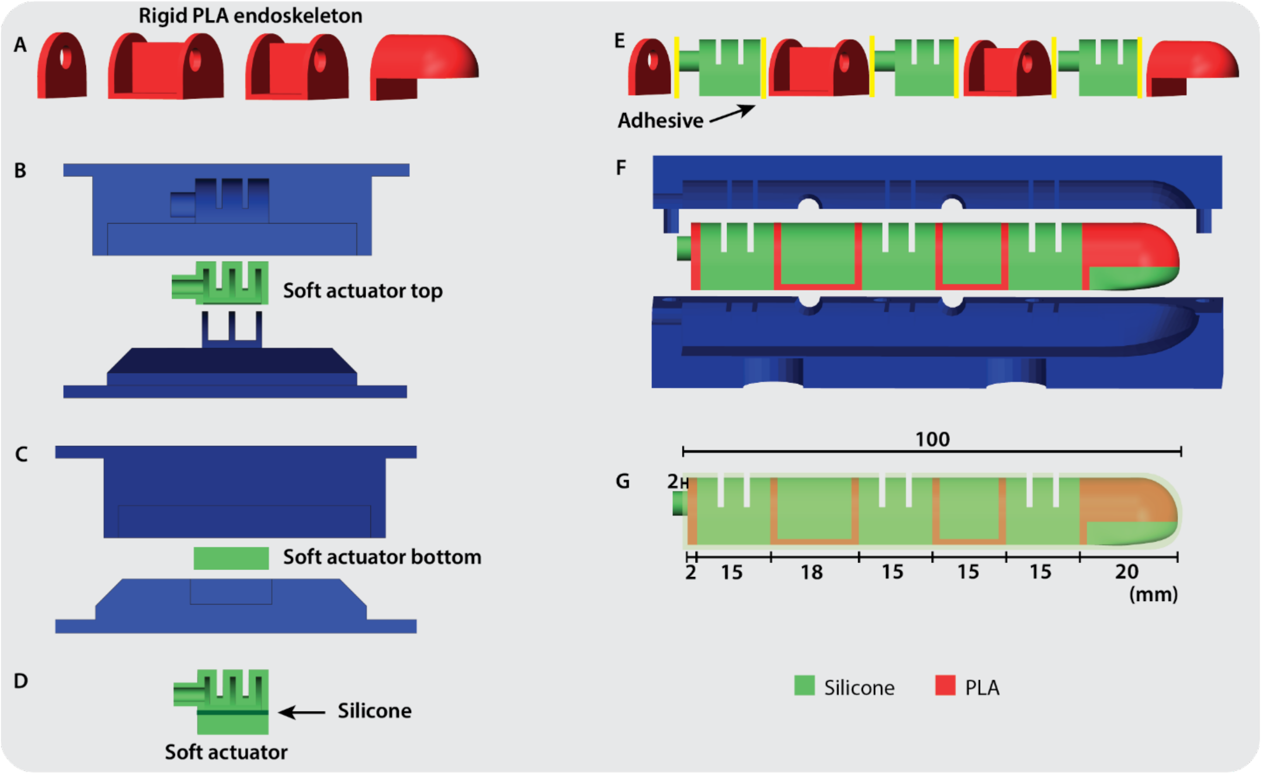

Fig. S1. Hybrid finger fabrication overview. (**A**) The Rigid pieces are 3D printed out of PLA and set aside. (**B, C**) The molds for the soft actuator are also 3D printed out of PLA. Dragon Skin™ 10 silicone is poured into the molds and cured for 20 minutes in a low temperature oven or open overnight. (**D**) Once the soft actuator's top and bottom are cured and removed from the molds, additional Dragon Skin™ 10 silicone is spread around the edges to seal the actuator halves together. (**E**) The soft actuators and rigid pieces are adhered together using clear silicone waterproof sealant. The tubes are inserted into the soft actuators through the rigid pieces and are sealed with silicone sealant and left to dry overnight. (**F**) Finally, the entire hybrid finger, except the valleys of the actuators, is coated with 2 mm of Dragon Skin™ 10 silicone in a 3D printed mold. (**G**) Final dimensions of the fabricated hybrid finger.

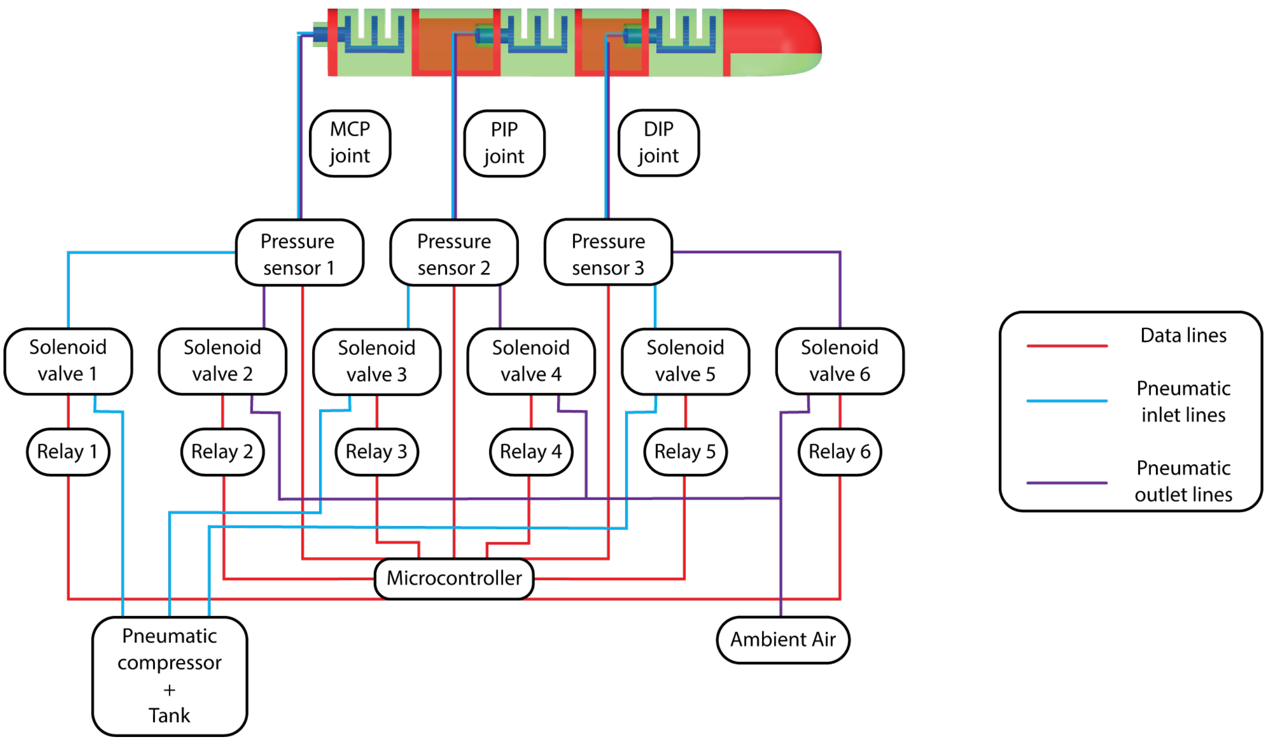

Fig. S2. Overview of the pneumatic system to actuate the hybrid finger. The scalable pneumatic system independently actuated each joint. It used an air compressor, six 2-way bidirectional miniature solenoid valves (Parker X-7-12-L-F), and pressure sensors (Honeywell ASDXACX100PAAA5) to regulate the airflow into each joint. However, this system can be simplified to use only three three-way direct-acting solenoid valves to regulate the air entering the joints from the compressor and releasing the air from the finger into the ambient atmosphere. An Arduino UNO microcontroller independently controls the pneumatic circuit. Relays are used to control the 12V valves using the microcontroller.

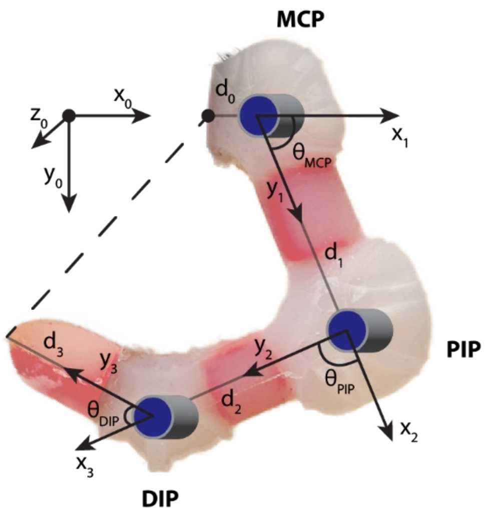

Fig. S3. Simplified kinematic model of the hybrid finger. The human finger inspired the design of the hybrid biomimetic finger and follows a similar kinematic model. The metacarpophalangeal (MCP), proximal interphalangeal (PIP), and distal interphalangeal (DIP) joints are pivot joints that create flexion through bending and straightening movements around a single axis.

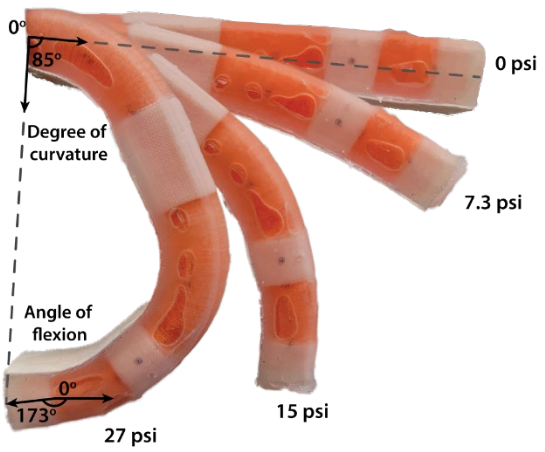

Fig. S4. Soft finger actuation for comparison. Side view of the simultaneous actuation of both joints of the pneumatically actuated soft finger at varying pressures when mounted horizontally on the UR5 robot arm.

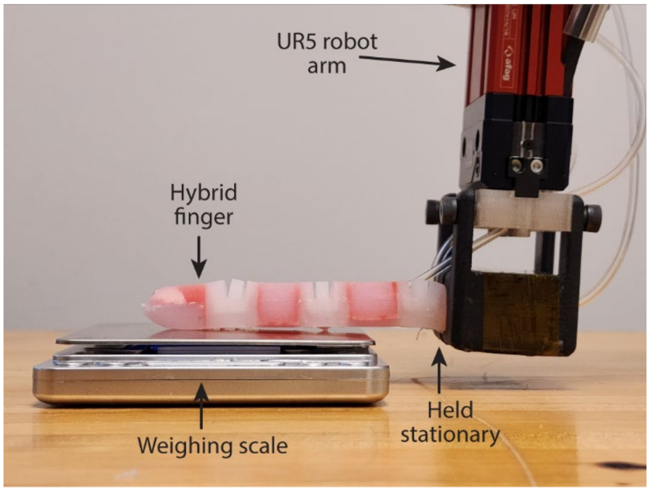

Fig. S5. Flexion force experimental setup. The hybrid and soft fingers were held stationarily by a UR5 arm above a weighing scale. All joints of the fingers were simultaneously actuated to the set pressures. No additional force was applied to the fingers.

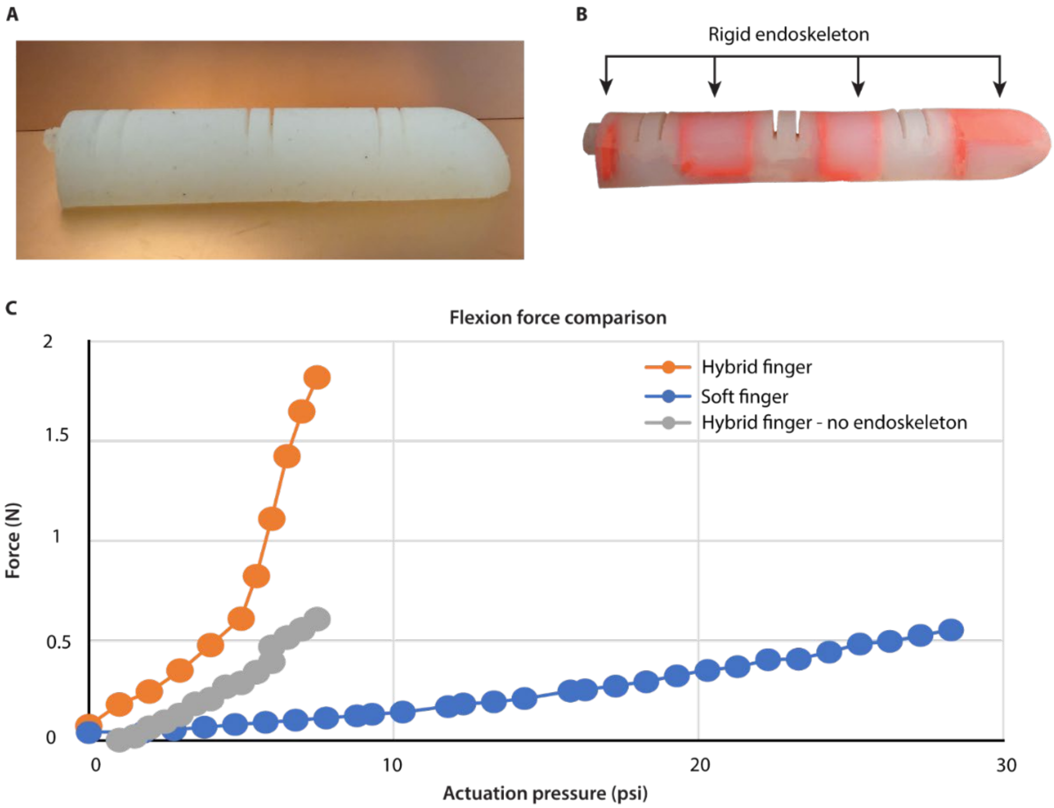

Fig. S6. Flexion force comparison using a hybrid finger with no rigid endoskeleton. (**A**) Hybrid finger with no rigid endoskeleton; (**B**) Hybrid finger with endoskeleton. (**C**) Flexion force comparison shows that the maximum force the hybrid finger with no endoskeleton could achieve was 0.61 N when actuated to 7 N, much lower than the hybrid finger with an endoskeleton that can achieve 1.8 N. Overall, the hybrid finger with no rigid endoskeleton was around the flexion force of the soft robotic finger (0.55 N), with a lower actuation pressure. Therefore, the rigid endoskeleton adds to the flexion force of the hybrid finger.

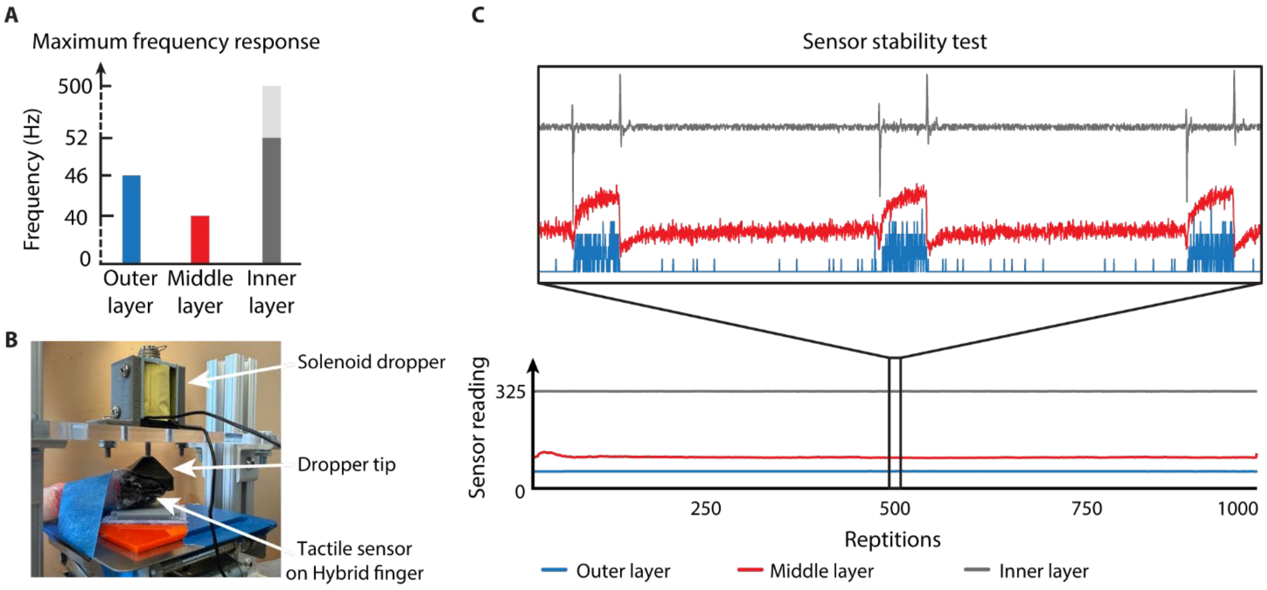

Fig. S7. Additional sensor characterization. (**A**) Maximum frequency response of each sensing layer of the multilayered tactile sensor indicating the transition from discrete to continuous. The Outer layer reached 46 Hz, the middle sensing layer reached 40 Hz, and the inner piezoelectric sensing layer stayed discrete at 52 Hz. The testing setup was only capable of reaching 52 Hz. Therefore, a Fast Fourier transform (fft) of the inner sensor layer showed a high magnitude up to 500 Hz, which is half our tactile sensing systems sampling rate of 1000 Hz. (**B**) The stability of the tactile sensor responses was measured by continuously dropping a tip on the tactile sensor at a constant force using a solenoid dropper 1000 times. (**C**) The average peak forces for each sensing layer are shown in the figure with a zoomed view of three repetitions. The multilayered tactile sensor was stable for 1000 repetitions, with minor fluctuations in the middle sensing layer at the beginning and end.

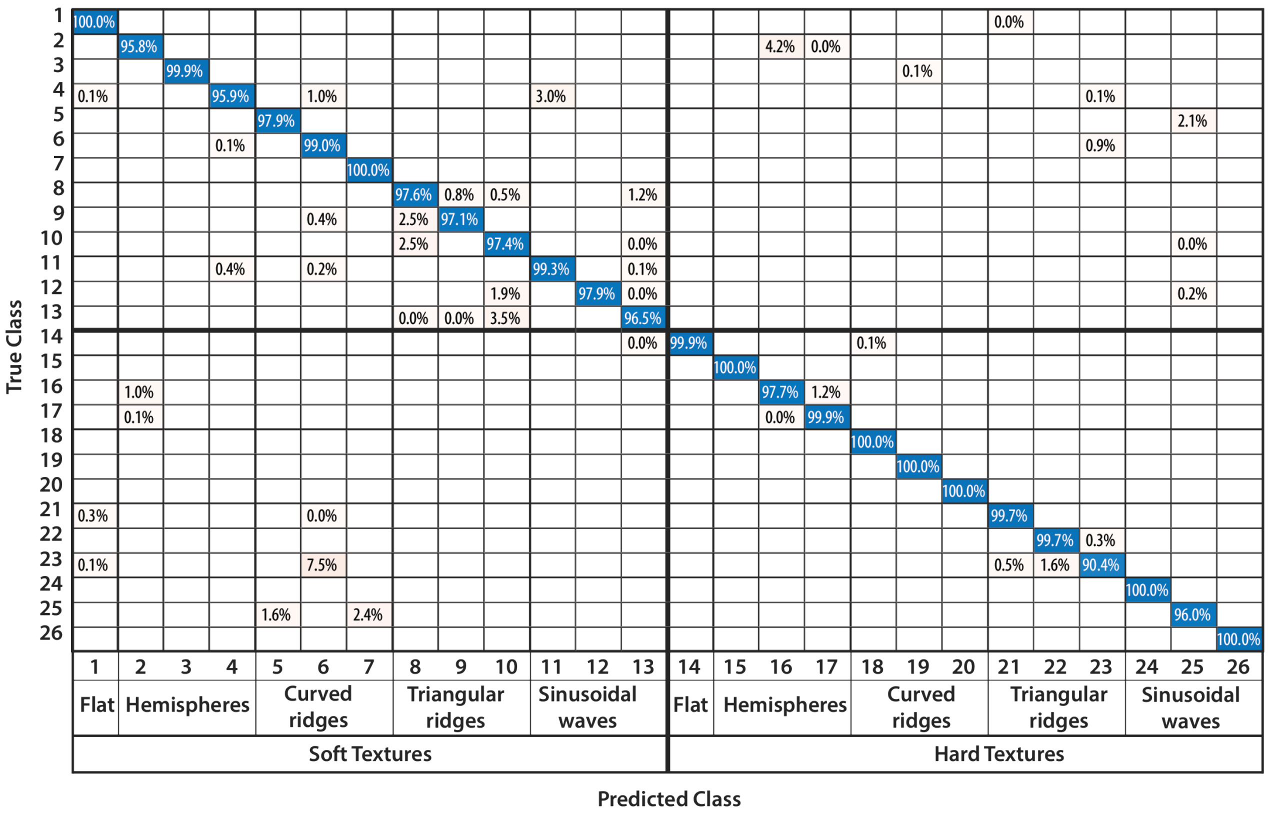

Fig. S8. Confusion matrix for texture classification with the multilayer sensor on the hybrid finger. The hybrid finger achieved a classification accuracy of 98.38%.

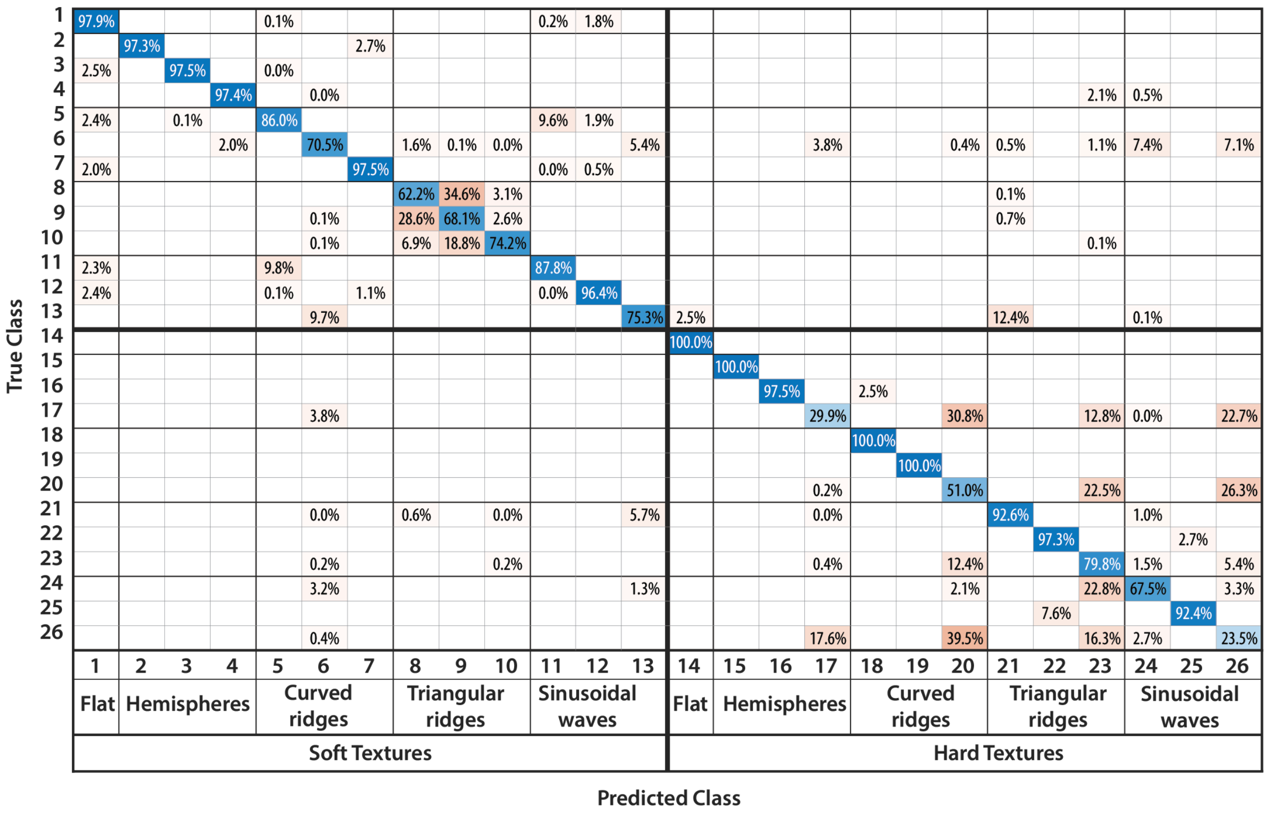

Fig. S9. Confusion matrix for texture classification with the soft finger. The soft finger had an average classification accuracy of 82.31% but had trouble differentiating the soft triangular ridges and hard textures with six textural repeats.

True Class	1	2	3	4	5	6	7	8	9	10	11	12	13	14	15	16	17	18	19	20	21	22	23	24	25	26
1	97.5%	0.7%	1.8%																							
2	1.1%	93.2%	0.1%	2.5%		2.6%										0.1%		0.3%				0.0%				
3	0.2%	4.1%	90.5%			2.5%	2.7%																			
4	2.3%	2.5%		92.9%												0.0%				0.1%			2.2%			
5					95.8%			0.4%	0.0%		0.1%	2.8%	0.9%													
6		1.7%	0.5%	0.0%		95.4%	0.1%																2.3%			
7			1.9%	0.0%		0.1%	95.7%																2.2%			
8					0.7%			75.9%	3.5%	2.2%	6.8%	8.3%	0.1%										0.4%		0.7%	1.6%
9								6.9%	66.3%		5.1%	0.0%	20.8%											0.8%		
10								0.2%		68.6%					0.0%	10.1%		2.1%		4.6%	0.7%	9.1%	2.1%			2.5%
11					0.0%			3.2%	1.5%		91.4%	3.6%	0.3%													
12					2.1%			5.9%			3.4%	86.4%	2.2%	0.0%												
13					2.4%			0.1%	6.8%		0.1%	2.5%	66.5%											21.6%		
14					0.0%									98.8%					1.2%							
15						0.3%	0.0%								76.4%	0.3%	0.1%	0.2%		15.8%	0.0%		5.2%			1.5%
16	2.6%			0.1%						6.3%					0.0%	78.2%	1.8%	0.8%		4.1%	0.6%	1.4%	4.1%			
17															1.7%	0.0%	90.4%	0.3%		5.5%	0.0%					2.1%
18															0.0%	0.1%	1.1%	79.8%		14.4%	4.6%					
19					2.5%														97.5%							
20				0.1%			0.1%	0.1%		5.4%					5.0%	7.1%	8.6%	6.3%		57.0%	4.9%	2.8%	2.7%			
21							0.3%								0.8%	0.6%		6.9%		3.0%	40.0%	45.5%	3.0%			
22										11.9%						1.6%		0.0%		1.7%	0.1%	84.6%				
23						0.0%		0.1%		3.1%					1.6%						4.6%	2.5%	81.8%			6.2%
24									0.1%				22.5%											77.4%		
25											0.0%	1.6%													98.4%	
26						0.4%									2.3%	0.0%							16.3%			81.0%
	Flat	Hemispheres			Curved ridges			Triangular ridges			Sinusoidal waves			Flat	Hemispheres			Curved ridges			Triangular ridges			Sinusoidal waves		
	Soft Textures													Hard Textures												

Predicted Class

Fig. S10. Confusion matrix for texture classification with the rigid finger. The rigid finger had a similar average classification accuracy of 83.02% and especially struggled with the soft and hard triangular ridges and soft sinusoidal waves.

True Class	1	2	3	4	5	6	7	8	9	10	11	12	13	14	15	16	17	18	19	20	21	22	23	24	25	26
1	97.7%			0.0%		0.7%	0.1%				0.0%										0.1%		1.4%			
2	0.1%	77.1%		2.9%				0.0%	3.3%	1.2%	2.7%	0.3%			0.9%				1.6%	1.7%	5.3%			2.8%		
3			89.0%								0.0%				1.9%	2.9%	1.3%		1.4%		1.7%			1.8%		
4	0.0%	1.7%		89.7%	0.3%	1.1%		0.1%		0.1%	5.0%		0.4%			0.0%							0.3%	1.2%		
5	0.0%				86.9%		2.5%															2.4%	2.0%		0.3%	6.0%
6				1.4%		87.5%							4.3%										6.0%		0.8%	
7	1.4%				5.0%		82.5%															7.2%	1.3%		2.6%	
8		0.0%				1.1%		78.9%	13.5%	2.8%			2.1%												1.7%	
9		1.1%						16.5%	77.5%	4.9%		0.0%														
10								1.8%	1.3%	96.9%																
11		4.0%		2.8%		0.3%					92.9%							0.1%								
12		0.0%							2.0%	1.2%		96.8%								0.1%						
13						2.4%			0.0%	3.8%			93.8%													
14														98.4%				1.6%								
15			1.8%	0.0%											81.2%	1.6%	3.1%		0.1%					12.1%		0.0%
16		1.9%										0.0%			3.6%	78.8%	8.8%		2.1%		2.3%			2.4%		
17	0.0%	0.4%	1.0%	2.2%								0.5%			3.1%	10.1%	74.6%		0.3%					6.5%		1.4%
18											2.8%							97.2%					0.0%			
19		0.0%													3.1%	6.8%	0.3%		82.8%		0.1%	0.0%		6.9%		
20				0.3%						1.0%		1.5%								97.2%						
21	0.3%	2.9%		0.0%			0.2%		0.2%		1.5%				0.0%	1.7%	0.0%		4.0%		89.2%					
22					1.3%		4.8%														1.8%	91.2%			0.9%	
23	1.5%				3.3%	4.2%	0.1%			1.8%			0.7%									0.0%	88.4%		0.0%	
24		0.2%	2.0%	0.6%								2.5%			10.4%	1.8%	2.3%		0.9%					79.2%		
25					0.3%		0.4%	1.5%	2.0%	0.1%		0.1%										0.3%	0.2%		92.3%	2.7%
26				2.4%	4.3%		0.0%										0.1%					0.7%			3.6%	88.9%
Predicted Class	1	2	3	4	5	6	7	8	9	10	11	12	13	14	15	16	17	18	19	20	21	22	23	24	25	26
	Flat	Hemispheres			Curved ridges			Triangular ridges			Sinusoidal waves			Flat	Hemispheres			Curved ridges			Triangular ridges			Sinusoidal waves		
	Soft Textures													Hard Textures												

Fig. S11. Confusion matrix for texture classification with only the outer sensing layer on the hybrid finger. The outer sensing layer performed well, with an 87.97% average classification accuracy.

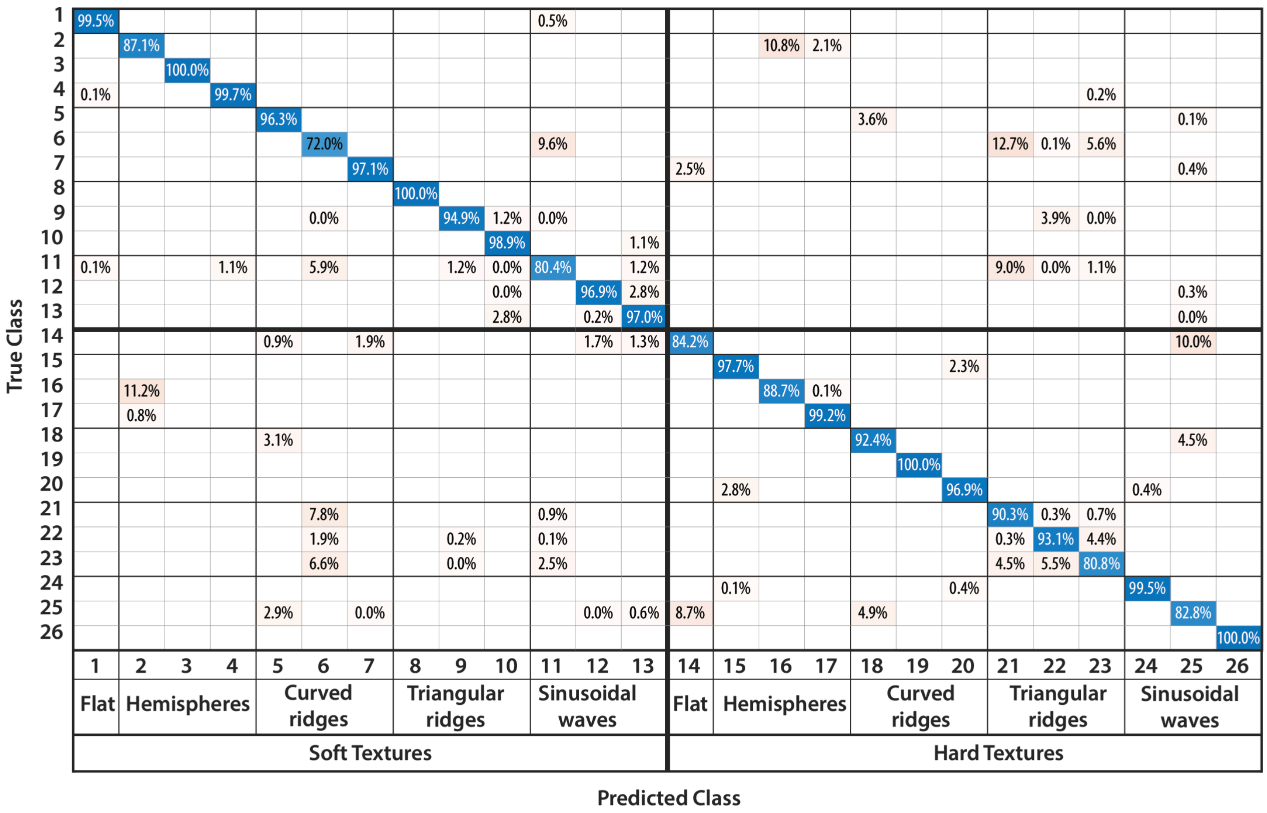

Fig. S12. Confusion matrix for texture classification with only the middle sensing layer on the hybrid finger. The middle layer performed best of the individual layers, with an average classification accuracy of 93.32%. This layer was best able to pick up the soft triangular ridges that the other sensing layers had difficulty with.

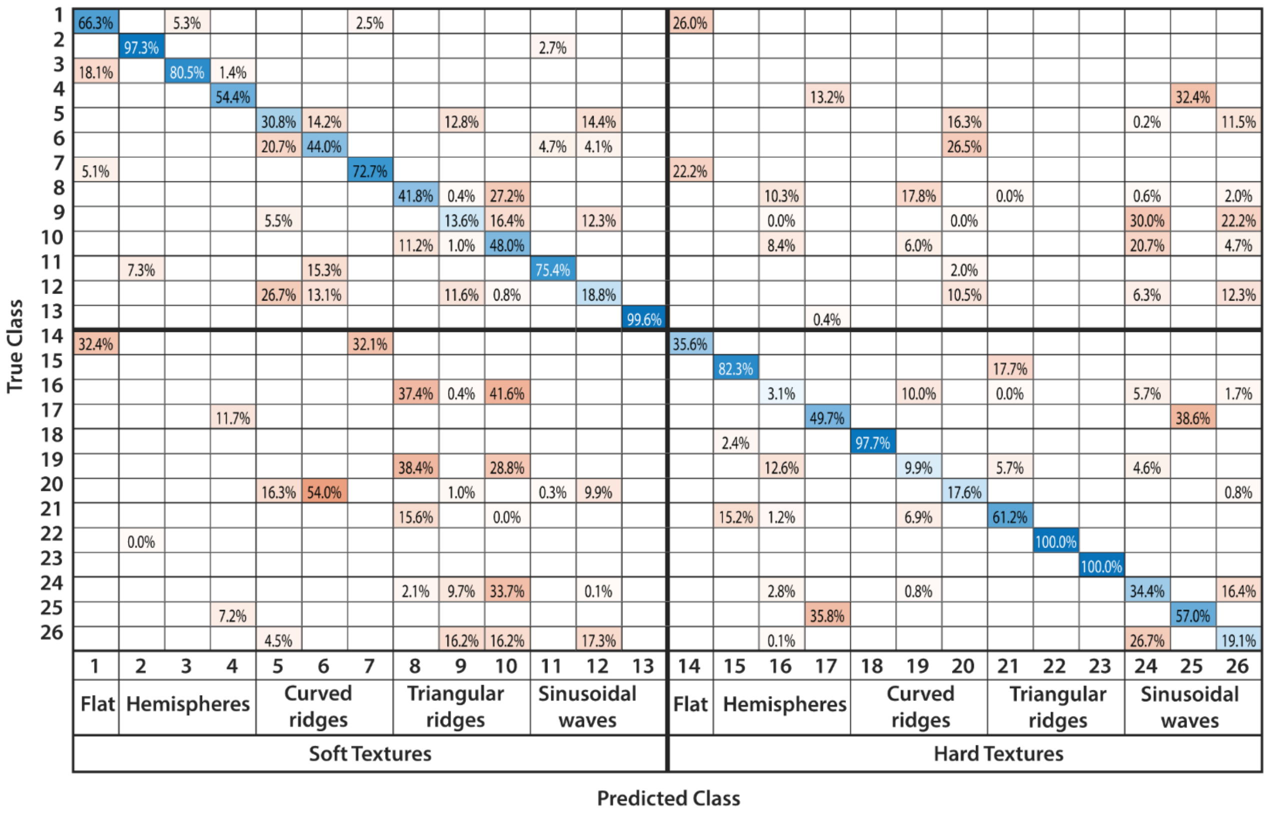

Fig. S13. Confusion matrix for texture classification with only the inner sensing layer on the hybrid finger. The inner piezoelectric sensor had difficulty with the soft textures, likely because they did not create as much vibration during palpation. It got an average classification accuracy of 54.21%.

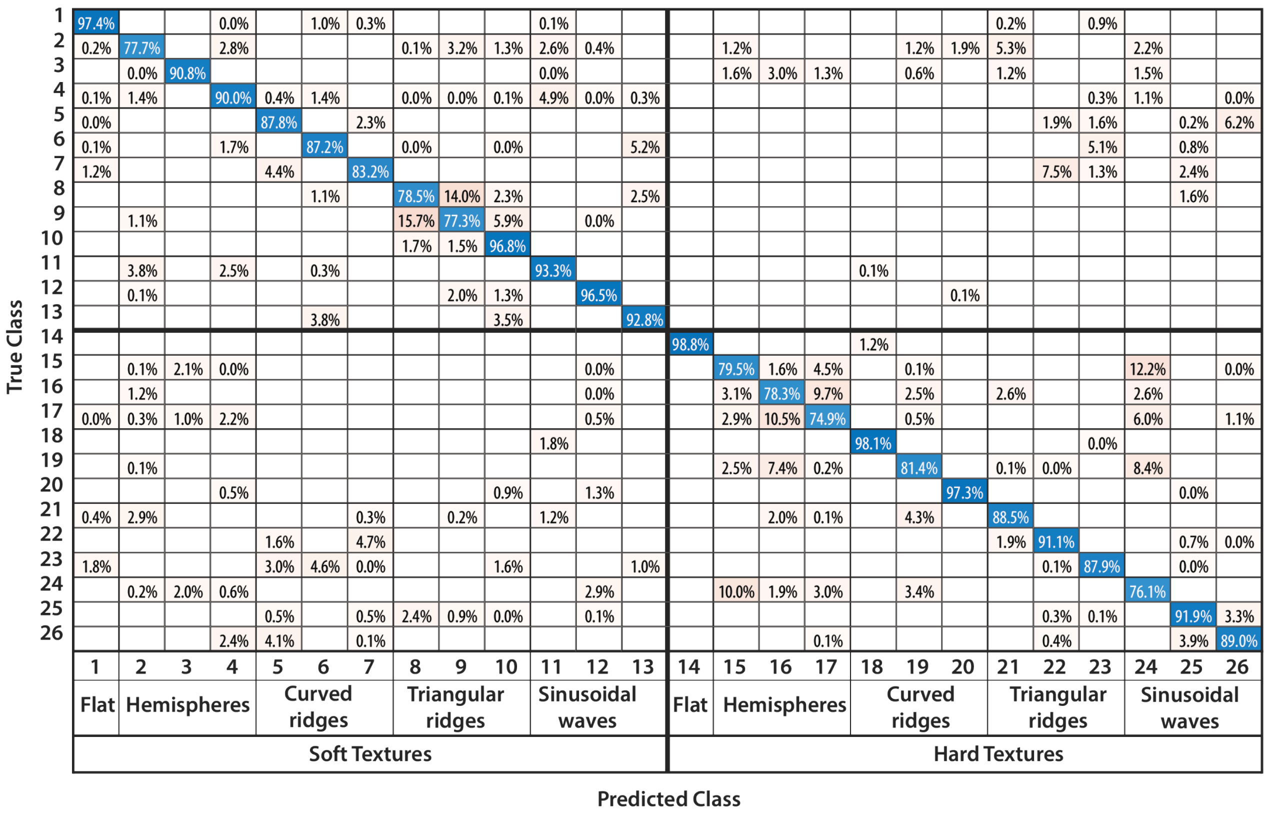

Fig. S14. Confusion matrix for texture classification with only the outer sensing layer with SA1 encoding on the hybrid finger. The outer sensing layer with only SA1 encoding performed well, with an 87.7% average classification accuracy. The accuracy is identical to the outer layer accuracy with both SA1 and FA1 encoding, meaning that RA1 encoding on the outer sensing layer does not bring in additional information from these textures.

True Class	1	2	3	4	5	6	7	8	9	10	11	12	13	14	15	16	17	18	19	20	21	22	23	24	25	26
1	70.5%	0.7%			1.9%	1.3%	17.2%												4.0%		2.0%	2.4%				
2	3.0%	47.4%		0.4%	1.4%	3.5%	4.7%	0.6%	5.9%		11.0%	2.8%			4.6%		2.2%		2.1%	3.2%	0.1%	0.1%	0.0%	7.0%	0.1%	
3			79.9%	0.5%	0.1%	0.0%									1.3%	4.1%	2.2%		2.9%		0.2%		2.8%	6.0%		
4			1.4%	77.3%	0.0%			0.6%		0.1%	5.0%		4.5%	0.3%	3.2%	2.9%	0.0%			0.0%				2.5%		2.1%
5			2.0%	2.0%	77.3%	8.3%	3.0%						0.1%				0.4%			0.6%			3.8%		1.4%	1.1%
6		7.6%			6.8%	63.7%	6.1%	2.3%	2.3%		0.5%	0.7%				0.0%			1.4%	0.9%		6.2%	0.1%		1.4%	
7	9.1%	0.7%					87.0%															0.1%	1.9%	0.0%	1.2%	
8		3.4%				0.1%		71.4%	16.0%	3.0%		3.5%	2.6%													
9		0.1%				0.1%		17.2%	72.0%	0.9%		9.6%												0.2%		
10								0.0%	5.1%	89.7%		4.4%	0.8%													
11		7.6%		0.4%	2.5%	0.2%					88.7%			0.5%												
12		1.1%			2.2%	0.1%		1.5%	24.1%	0.3%		69.4%												1.0%	0.2%	
13					0.3%	0.0%		2.7%	1.3%	1.4%		2.3%	91.9%											0.0%		
14											3.1%			90.8%				6.1%								
15	0.9%	3.2%	2.9%	2.5%											81.3%	0.8%	1.5%			0.0%	5.8%			0.2%		0.9%
16		0.2%	3.4%	0.1%		0.0%					0.0%				0.7%	75.2%	8.8%		2.8%		8.8%					0.0%
17	2.4%		3.0%	0.4%		0.0%							0.2%		7.6%	3.8%	74.7%			0.0%	5.3%			0.1%		2.5%
18											1.8%			2.1%				96.1%		0.0%						
19	0.9%		2.6%													0.1%			82.6%			9.7%	1.5%	2.5%		
20		2.6%	0.0%	0.1%		0.0%		0.2%	2.1%			0.3%	1.2%		0.2%					87.9%		0.0%	0.9%	4.0%	0.4%	0.0%
21	5.9%		0.4%				0.8%								2.6%	2.8%			0.1%	0.0%	87.2%	0.0%	0.0%			
22	4.4%						3.4%												13.7%		2.5%	65.6%	10.3%			
23	0.0%		4.7%			1.1%	2.8%												0.1%	2.5%		9.5%	75.1%		4.3%	
24	2.3%	13.1%	8.9%	2.8%			0.6%	0.0%	0.6%			0.1%	0.1%						0.1%	2.4%			0.8%	64.0%	4.2%	
25		0.0%		0.2%		3.0%	0.1%	0.1%	5.3%			1.9%	0.1%							2.5%		2.5%	7.0%	2.5%	74.7%	
26	2.7%	0.1%		1.0%											4.8%		9.8%			2.8%	0.0%					78.8%
	Flat	Hemispheres			Curved ridges			Triangular ridges			Sinusoidal waves			Flat	Hemispheres			Curved ridges			Triangular ridges			Sinusoidal waves		
	Soft Textures													Hard Textures												

Predicted Class

Fig. S15. Confusion matrix for texture classification with only the outer sensing layer with FA1 encoding on the hybrid finger. The outer sensing layer with only FA1 encoding did not perform as well, with a 77.69% average classification accuracy.

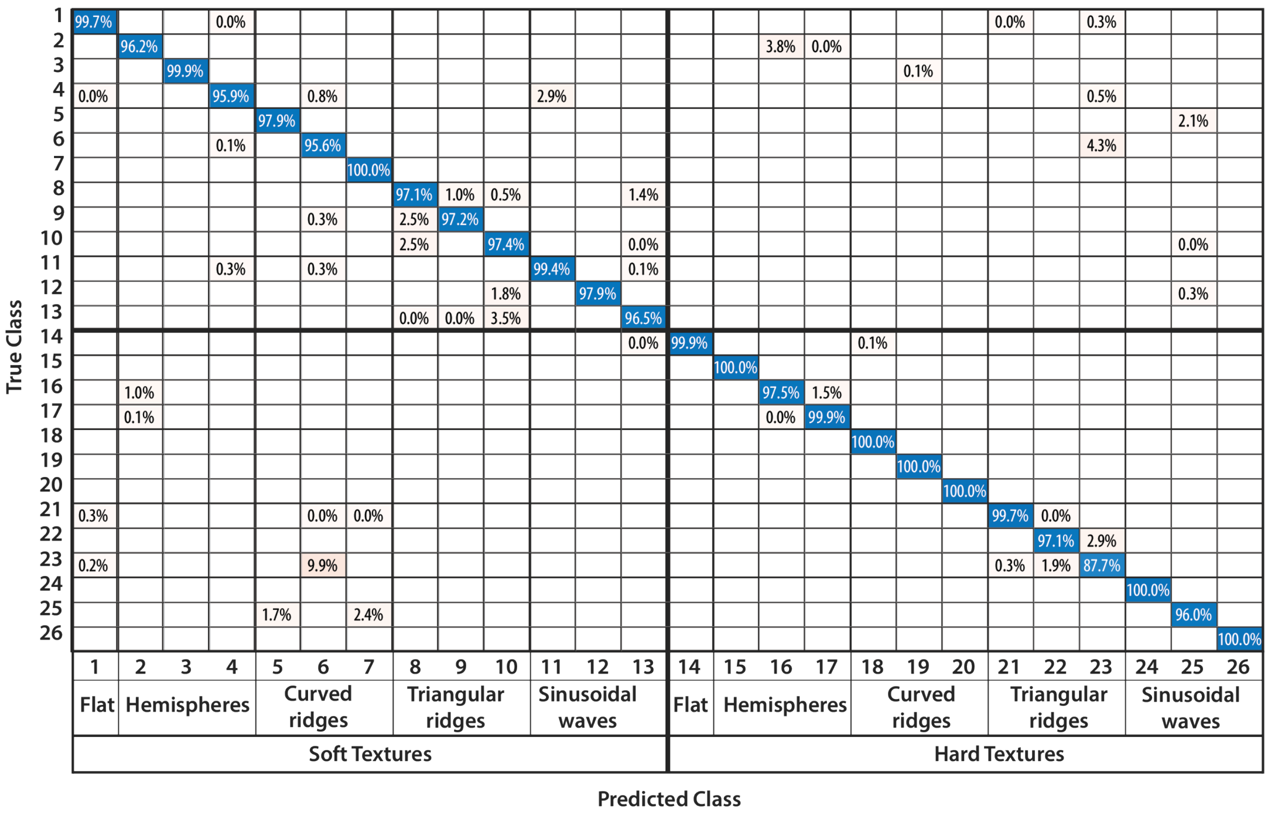

Fig. S16. Confusion matrix for texture classification with only the hybrid finger's outer and middle sensing layers. The sensing performance of both layers together was nearly that of the full multilayered sensor, with a 97.98% average classification accuracy. The result indicates that the outer and middle layers brought in varied information that increased the overall performance of the tactile sensing.

True Class	1	2	3	4	5	6	7	8	9	10	11	12	13	14	15	16	17	18	19	20	21	22	23	24	25	26
1	99.1%	0.0%		0.0%		0.5%	0.3%				0.0%										0.1%					
2	0.1%	77.4%		2.9%				0.0%	3.0%	1.2%	2.6%	0.3%			0.4%	0.0%			1.4%	2.2%	5.3%			3.2%		
3			90.5%												0.6%	2.1%	2.0%		1.1%		1.5%			2.1%		
4	0.0%	1.8%		89.5%	0.5%	1.5%		0.1%	0.0%	0.3%	5.1%	0.0%	0.3%											1.0%		0.0%
5	0.0%				91.0%		0.3%															2.5%			0.1%	6.1%
6	0.0%			1.3%		93.1%		0.0%		0.0%			4.3%										0.5%		0.8%	
7	1.5%				4.0%		86.9%															5.2%			2.5%	
8		0.0%				1.0%		79.1%	13.5%	2.6%			2.3%												1.3%	
9		1.1%						15.8%	78.4%	4.7%		0.1%														
10								1.9%	1.4%	96.7%																
11		4.1%		2.1%		0.3%					93.4%							0.0%								
12		0.0%							2.2%	1.5%		96.1%								0.1%						
13						2.3%				3.4%			94.2%													
14														98.9%				1.1%								
15			1.9%												82.3%	1.1%	2.2%		0.1%					12.5%		
16		1.9%										0.0%			4.6%	81.0%	5.4%		2.2%		1.9%			2.9%		
17		0.1%	1.6%	1.3%								0.4%			0.8%	5.7%	82.3%		0.2%					6.5%		1.1%
18											2.7%							97.3%					0.0%			
19		0.0%													2.5%	7.3%	0.0%		82.8%		0.1%			7.3%		
20				0.4%						0.9%		1.7%								97.0%				0.0%	0.0%	
21	0.2%	2.8%					0.2%		0.2%		1.5%					2.1%	0.0%		4.2%		88.7%					
22					1.4%		2.3%														1.8%	93.8%			0.8%	
23	0.7%				2.4%	2.0%				2.1%			0.2%										92.5%			
24		0.2%	1.8%	0.5%								2.5%			8.1%	1.7%	0.8%		1.5%					82.9%		
25					0.5%		0.2%	1.2%	2.3%	0.1%		0.2%										0.4%			93.0%	2.1%
26				2.4%	4.3%																	0.6%			2.4%	90.3%
	Flat	Hemispheres			Curved ridges			Triangular ridges			Sinusoidal waves			Flat	Hemispheres			Curved ridges			Triangular ridges			Sinusoidal waves		
	Soft Textures													Hard Textures												

Predicted Class

Fig. S17. Confusion matrix for texture classification with only the hybrid finger's outer and inner sensing layers. The sensing performance of both layers together was higher than each sensing layer independently, with an 89.64% average classification accuracy. The result indicates that the outer and inner layers brought in some varied information that increased the overall performance of the tactile sensing but not as much as the outer and middle layers or the middle and inner layers.

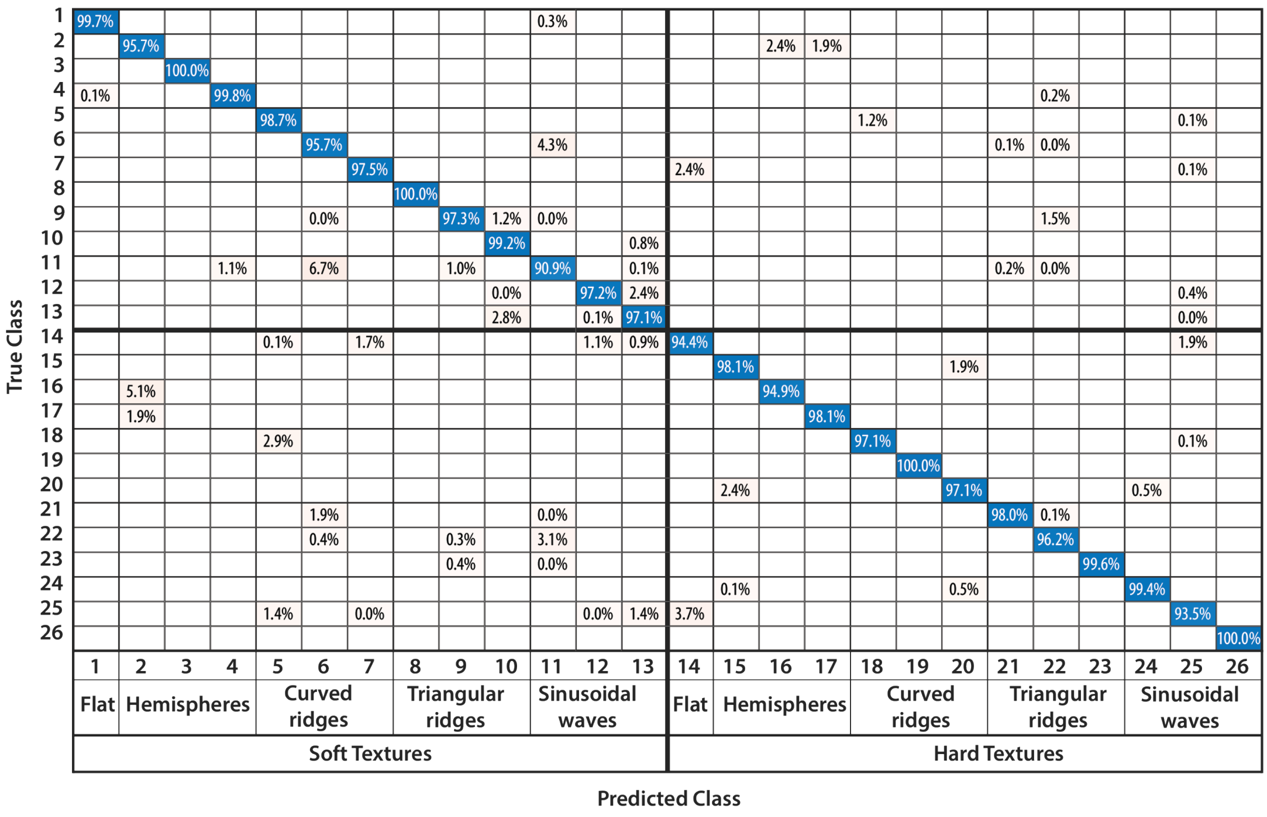

Fig. S18. Confusion matrix for texture classification with only the hybrid finger's middle and inner sensing layers. The sensing performance of both layers together was nearly that of the full multilayered sensor, with a 97.47% average classification accuracy. The result indicates that the middle and inner layers brought in varied information that increased the overall performance of the tactile sensing.

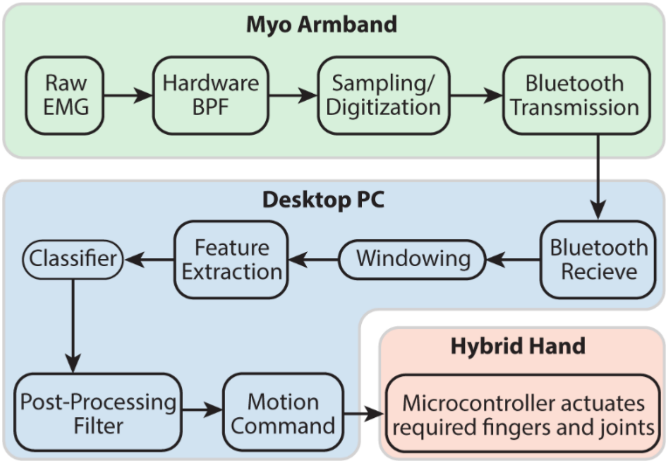

Fig. S19. EMG classification using the Myo Armband pipeline. The raw EMG signals are picked up by the electrodes on the armband, passed through the built-in Band Pass Filter (BPF), converted to digital signals, and sent over Bluetooth to the Desktop PC. Using the Myo Connect software, the EMG signals are windowed, and features are extracted for the classifier to differentiate the various hand positions. Once the hand position is determined by the classifier after a post-processing filter, the resulting motion command is sent to the microcontroller that actuates the required fingers and joints to match the intended hand position.

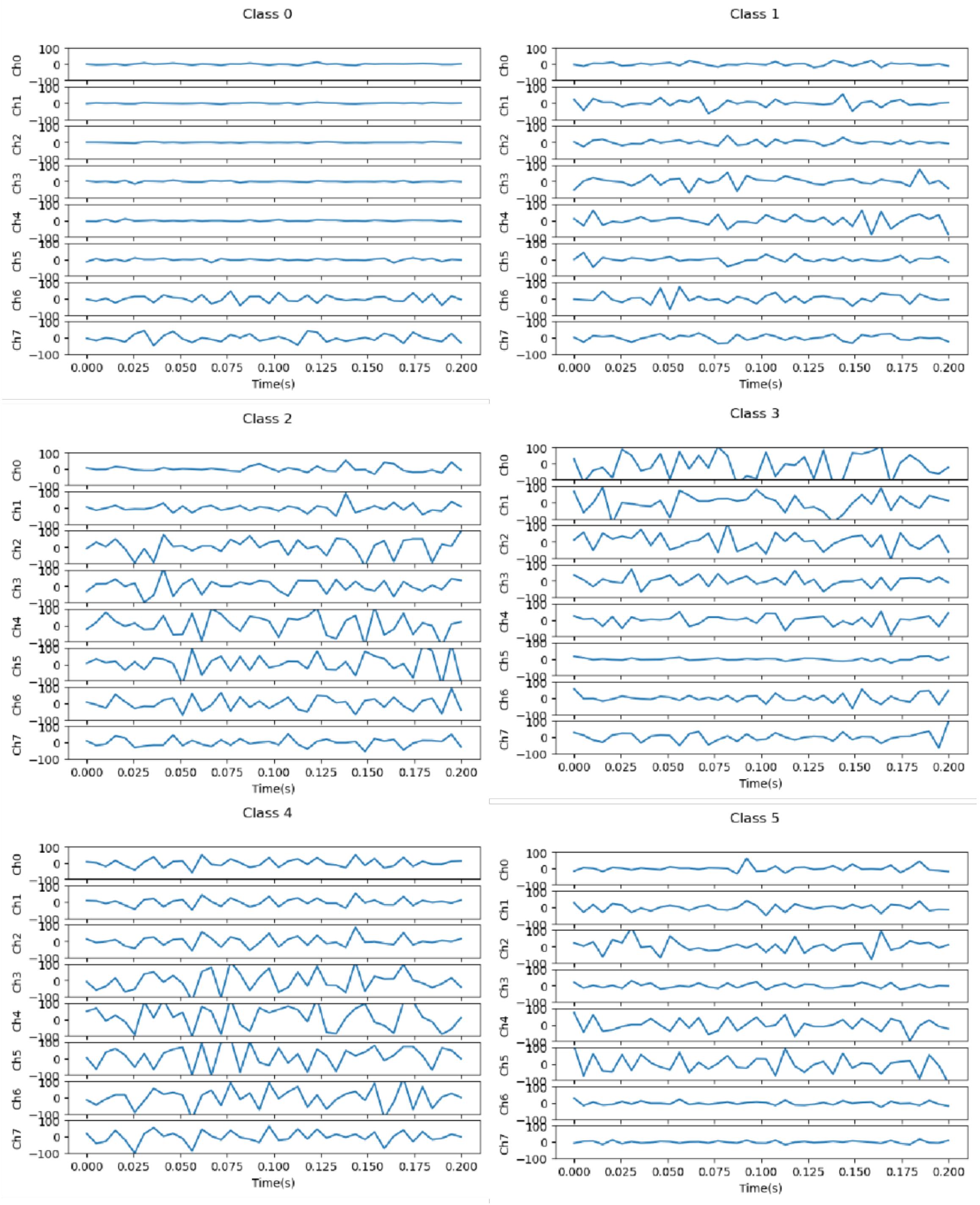

Fig. S20. Raw EMG signals from the Myo Armband for 6 classes of hand positions. The hybrid hand can have various hand positions with independently actuated joints on each finger. Six classes can easily be identified from the 8 electrode channels on the armband. These 6 classes cover the standard of hand positions performed by the hybrid hand and other myoelectric prosthetic hands.

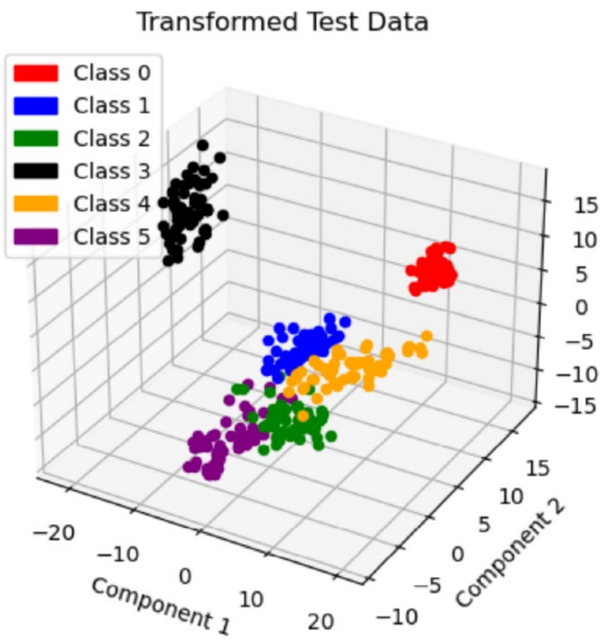

Fig. S21. Transformed Raw EMG signals from the Myo Armband in 3D feature space. Each color represents an individual class or hand position in the 3D feature space.

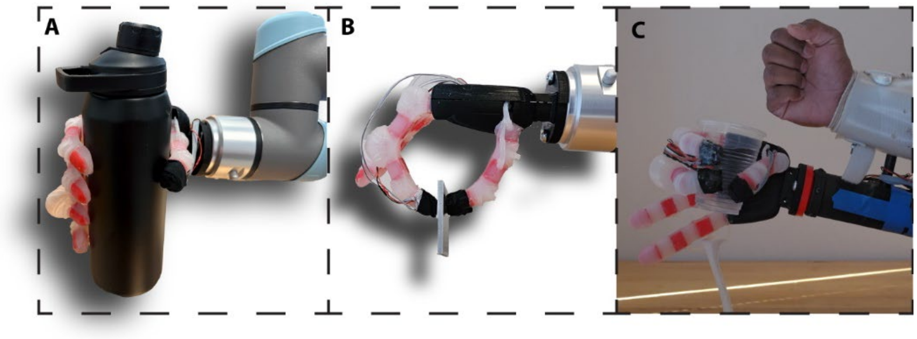

Fig. S22. Heavy object grasping capability of the hybrid hand. The hybrid hand grasping **(A)** a filled metal water bottle (1600g); (**B**) a heavy metal plate using the pinch grip with 2 fingers (143 g); (**C**) a fragile plastic cup filled with water using the tripod grip with 3 fingers (280 g).

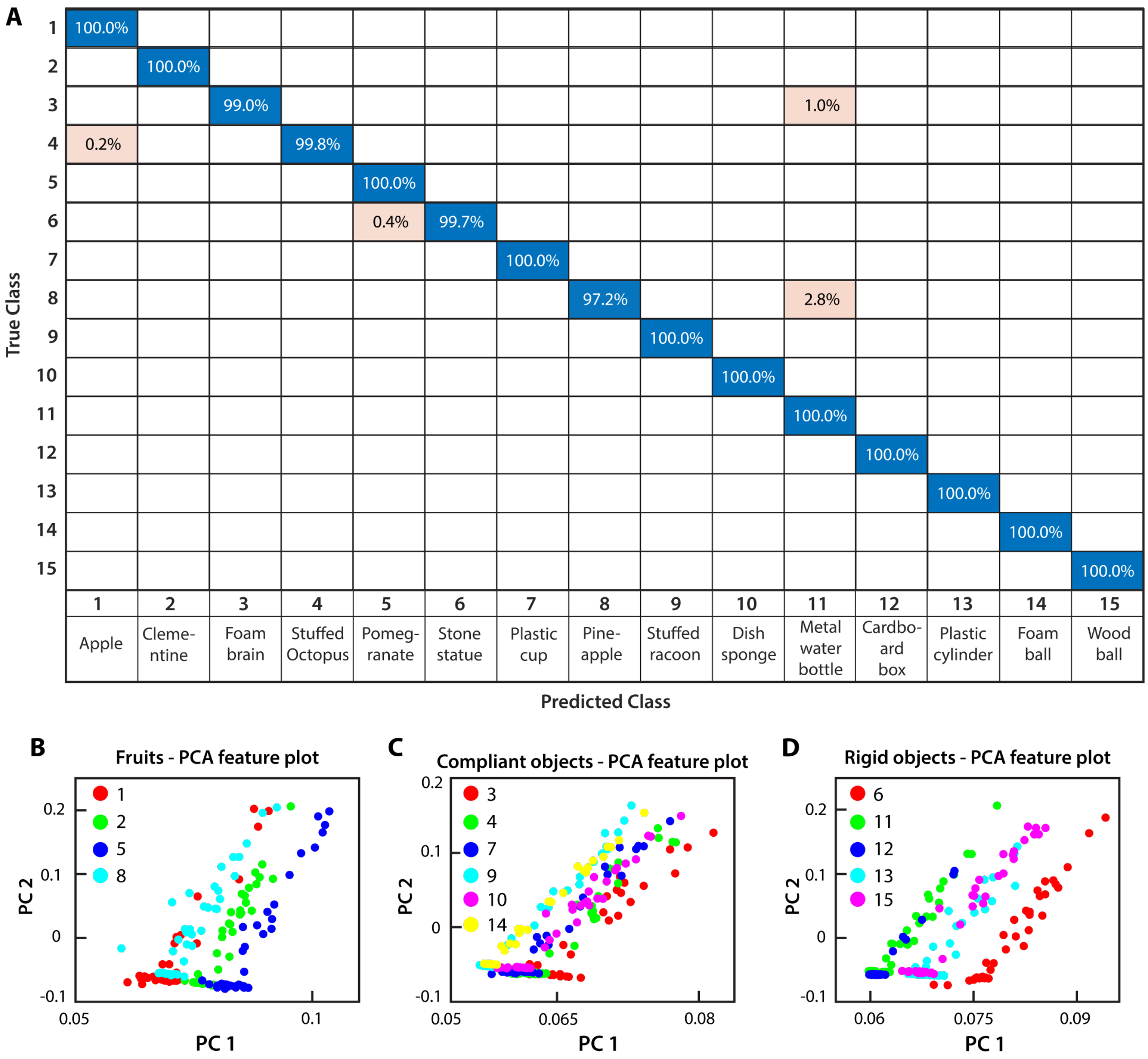

Fig. S23. Confusion matrix and PCA for everyday object differentiation during grasping. (A) The **c**onfusion matrix shows the only confusion came from the pineapple. The results indicate that each sensing layer brought varied textural information that increased the overall classification performance. (**B-D**) Visualization of the feature space when plotting the first 2 principal components based on the type of objects being grasped. (B) Fruits, (C) Compliant objects, and (D) Rigid objects show a good separation between objects of similar type of compliance.

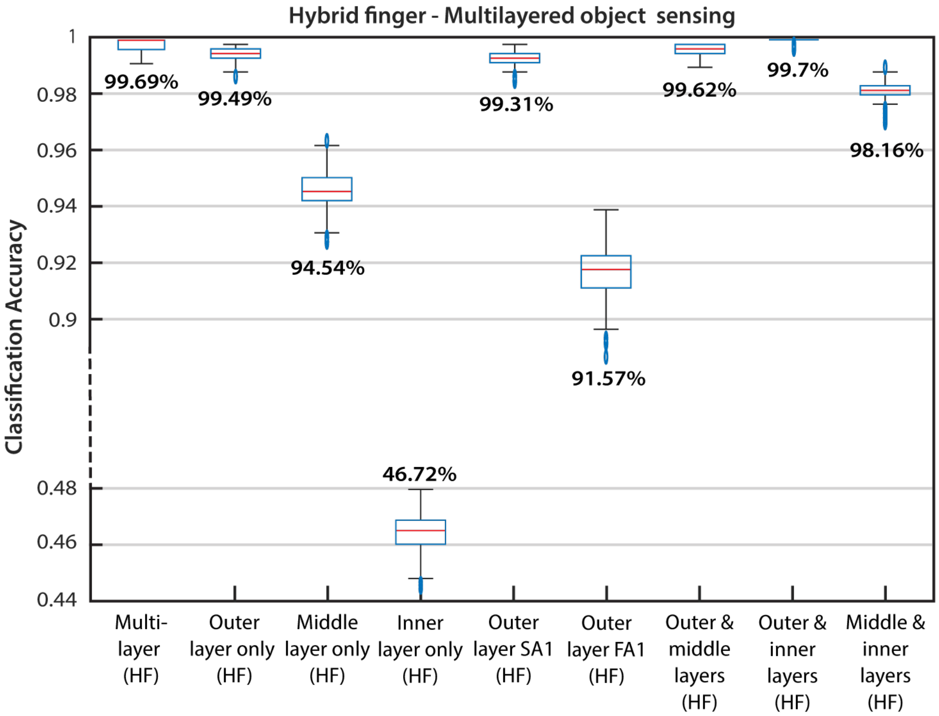

Fig. S24. Multilayered sensing for everyday object differentiation during grasping. The classification accuracy of the individual sensing layers of the hybrid finger and their combinations were also assessed. Overall, these everyday objects were not complex for the multilayered tactile sensor to differentiate while grasping. Most everyday objects do not have minor surface variations that require a sensor with high frequency or sensitivity to differentiate.

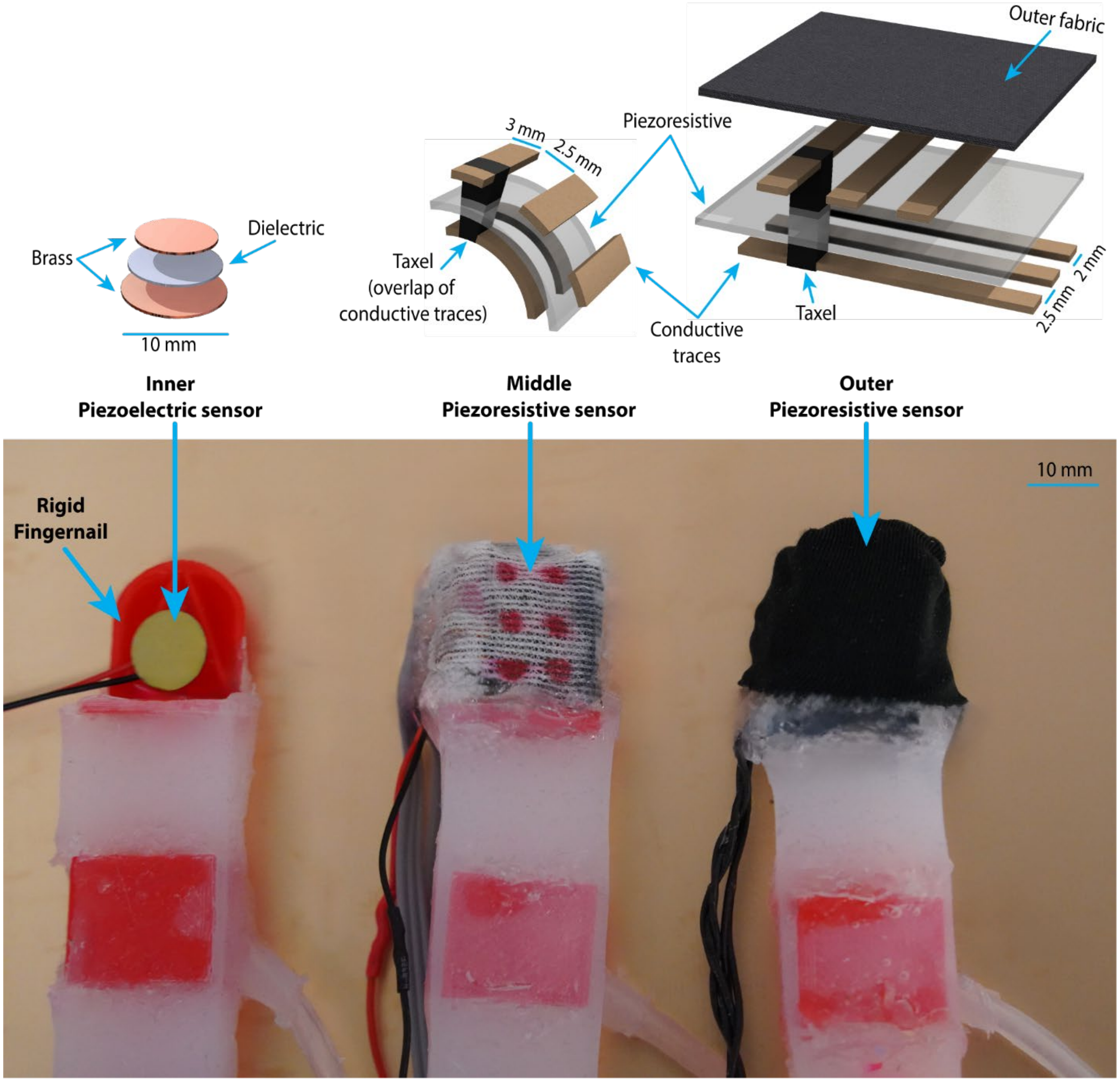

Fig. S25. Detailed view of the multilayered tactile sensor. Image of the three tactile sensing layers with exploded views of each layer.

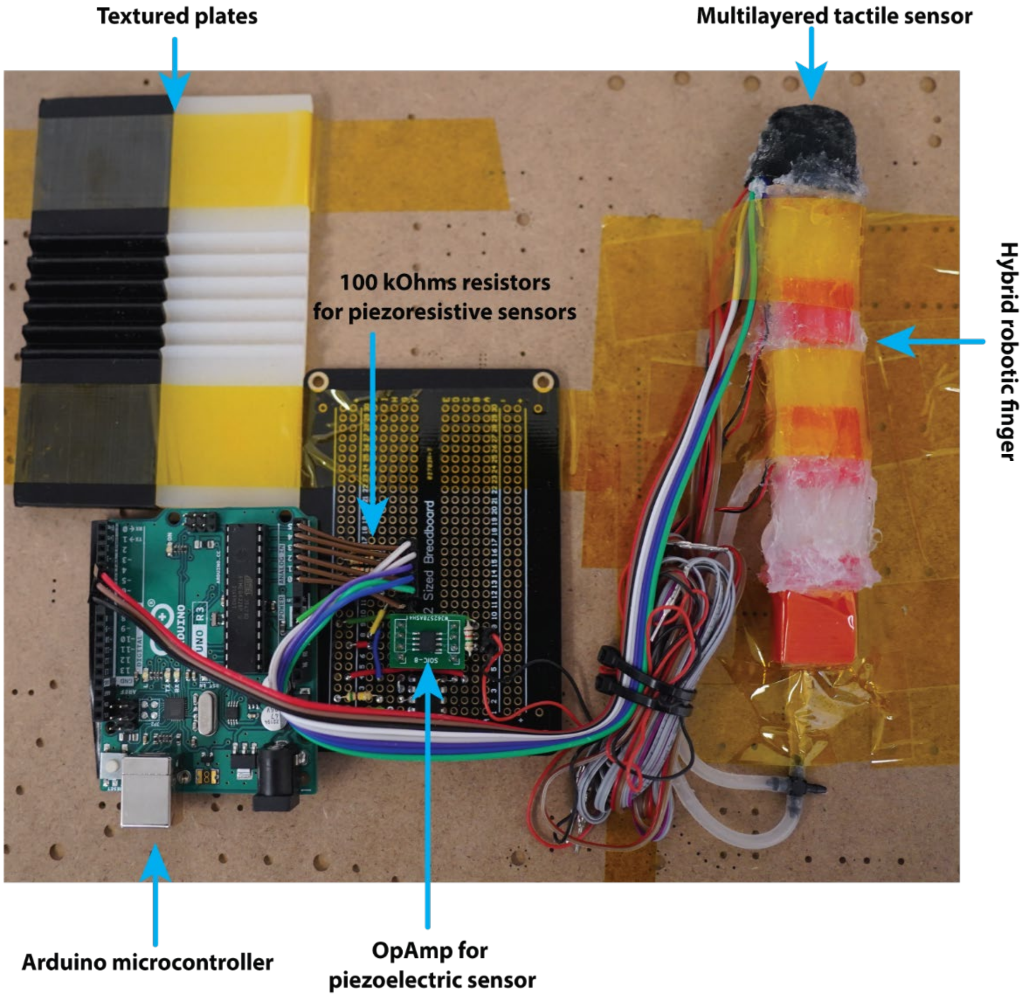

Fig. S26. Data collection setup of the hybrid finger. The sensor responses were collected at 1000 samples/sec sampling rate using an Arduino microcontroller.

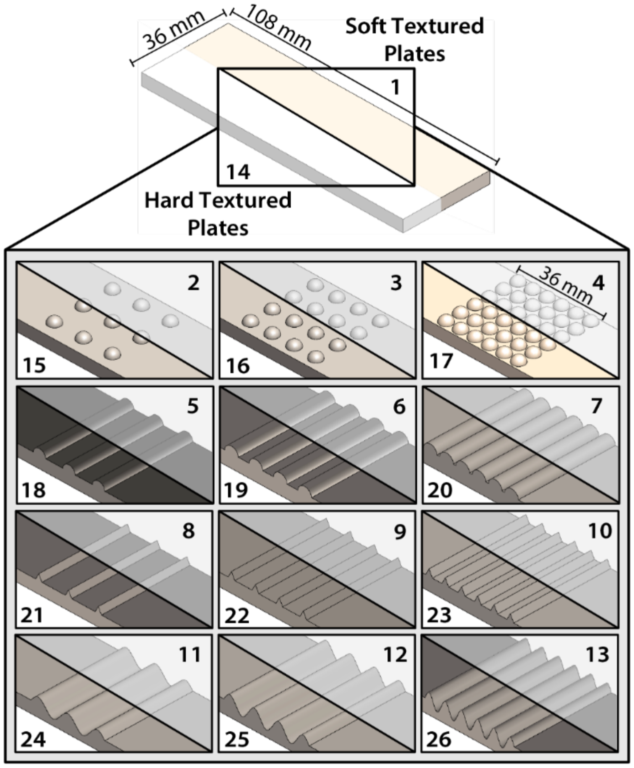

Fig. S27. Textured plates for texture discrimination. Twenty-six textured plates were designed to evaluate the hybrid finger's ability to discriminate textures. Two sets of thirteen textured plates, one was hard made of PLA and the other was soft made of silicone. There are four textural elements shared between the hard and soft textured plates, flat (soft:1, hard:14) hemispheres (soft:2-4, hard:15-17), curved ridges (soft:5-7, hard:18-20), triangular ridges (soft:8-10, hard:21-23), and sinusoidal waves (soft:11-13, hard:24-26). Each textural element varied between 3, 4, and 6 repeats in the 36x36 mm^2 textured surface.

Table S1. Flexion force for individual joints of the Hybrid finger at 7 psi

	Actuation pressure (psi)	Weight (g)	Force (N)
**All joints**	7	185.5	1.82
**MCP**	7	77.8	0.76
**PIP**	7	65.5	0.64
**DIP**	7	55.4	0.54
**MCP + PIP**	7	145.3	1.43
**PIP + DIP**	7	101.12	0.99
**MCP + DIP**	7	113.29	1.11

Table S2. ANOVA results comparing the hybrid finger's texture classification accuracy versus the soft and rigid fingers

	Mean squared error (MSE)	Root mean squared error (RMSE)	Sum of squares of the error (SSE)	Sum of squares regression (SSR)	Total sum of squares (SST)	Coefficient of determination (R^2)	F-statistic
**Hybrid vs. soft finger**	$1.830e^{-5}$	0.0043	0.0366	12.9149	12.9514	0.9972	$7.057e^{5}$
**Hybrid vs. rigid finger**	$2.50e^{-5}$	0.005	0.0501	11.7926	11.8427	0.9958	$4.706e^{5}$
**Hybrid (outer layer) vs. soft finger**	$3.53e^{-5}$	0.0059	0.0706	1.603	1.673	0.9578	$4.54e^{4}$
**Hybrid (outer layer) vs. rigid finger**	$4.207e^{-5}$	0.0065	0.0841	1.224	1.308	0.9357	$2.91e^{4}$

Movie S1. Texture palpation - Sensing and Encoding. The fingers are mounted on the UR5 arm and brought over the textures to palpate them. The hybrid finger runs were unique because the finger's angle of flexion required additional waypoints to extend the finger before palpation. The resulting tactile sensor response and neuromorphically encoded spiking activity are displayed.

Movie S2. Hybrid robotic hand actuation. The actuation of the hybrid robotic hand and each hybrid finger are shown.

Movie S3. Object grasping on the UR5 robotic arm. The hybrid robotic hand is shown gasping and placing a ball, followed by a handshake with a human hand.

Movie S4. Electromyography (EMG) control of the Hybrid robotic hand on a bypass prosthesis. People with upper limb loss traditionally use EMG to control myoelectric prosthetic hands. In this video, we show that the hybrid robotic hand controlled through EMG with a bypass prosthesis can grasp compliant everyday objects. The bypass prosthesis allows able-bodied subjects to use a prosthetic hand in experiments.
